# Enhanced Slime Mould Algorithm for Multilevel Thresholding Image Segmentation Using Entropy Measures

**DOI:** 10.3390/e23121700

**Published:** 2021-12-20

**Authors:** Shanying Lin, Heming Jia, Laith Abualigah, Maryam Altalhi

**Affiliations:** 1 College of Marine Engineering, Dalian Maritime University, Dalian 116026, China; 2 School of Information Engineering, Sanming University, Sanming 365004, China; 3 Faculty of Computer Sciences and Informatics, Amman Arab University, Amman 11953, Jordan; aligah.2020@gmail.com or; 4 School of Computer Science, Universiti Sains Malaysia, Pulau Pinang 11800, Malaysia; 5 Department of Management Information System, College of Business Administration, Taif University, P.O. Box 11099, Taif 21944, Saudi Arabia; marem.m@tu.edu.sa

**Keywords:** multilevel thresholding image segmentation, slime mould algorithm, minimum cross-entropy, meta-heuristics

## Abstract

Image segmentation is a fundamental but essential step in image processing because it dramatically influences posterior image analysis. Multilevel thresholding image segmentation is one of the most popular image segmentation techniques, and many researchers have used meta-heuristic optimization algorithms (MAs) to determine the threshold values. However, MAs have some defects; for example, they are prone to stagnate in local optimal and slow convergence speed. This paper proposes an enhanced slime mould algorithm for global optimization and multilevel thresholding image segmentation, namely ESMA. First, the Levy flight method is used to improve the exploration ability of SMA. Second, quasi opposition-based learning is introduced to enhance the exploitation ability and balance the exploration and exploitation. Then, the superiority of the proposed work ESMA is confirmed concerning the 23 benchmark functions. Afterward, the ESMA is applied in multilevel thresholding image segmentation using minimum cross-entropy as the fitness function. We select eight greyscale images as the benchmark images for testing and compare them with the other classical and state-of-the-art algorithms. Meanwhile, the experimental metrics include the average fitness (mean), standard deviation (Std), peak signal to noise ratio (PSNR), structure similarity index (SSIM), feature similarity index (FSIM), and Wilcoxon rank-sum test, which is utilized to evaluate the quality of segmentation. Experimental results demonstrated that ESMA is superior to other algorithms and can provide higher segmentation accuracy.

## 1. Introduction

Image segmentation is fundamental and challenging work in computer vision, pattern recognition, and image processing. It is widely used in various fields, such as ship target segmentation and medical image processing [1]. The main goal of segmentation is to divide the image into homogeneous classes. The elements of each class share common attributes such as grayscale, feature, color, intensity, or texture [2,3,4,5]. In the literature, there are four standard image segmentation methods, which can be divided into (1) clustering-based methods, (2) region-based methods, (3) graph-based methods, (4) thresholding-based methods. Among the existing methods, one of the most widespread techniques is multilevel thresholding, which is widely used owing to its ease of implementation, high performance, and robustness compared with other methods [6]. Image thresholding techniques can be classified into two categories: Bilevel and multilevel. In the prior category, the image is separated into two homogeneous foreground and background areas using a single threshold value. The latter segment-techniques segment divides an image into more than two regions based on pixel intensities known as histogram [7]. Bilevel thresholding can solve simple image segmentation problems involving only two grey levels. However, the bilevel cannot be suitable for complicated and high-grade images. Therefore, the multilevel thresholding technique is the primary method for real-world applications [8]. Generally speaking, selecting threshold values is crucial when segmenting an image because of the enormous image thresholds. Consequently, it is formulated into an optimization problem, which includes parametric or nonparametric methods [9].

The parametric approach considers that each image class can be defined using probability density distributions, but this technique is computationally expensive. By contrast, the nonparametric approach uses criteria to separate the pixels into homogeneous regions, and then the thresholds are determined using statistical measures (entropy or variance) [10]. Over the years, many works in the literature have proposed some of these criteria. Among them, Otsu’s technique maximizes the between-class variance of each segmented class to achieve the optimal thresholds [11]. Kapur’s approach used the entropy of the histogram as a formula to obtain the optimal thresholds [12]. Li et al. [13] presented the minimum cross-entropy to minimize the cross-entropy between the original and segmented image to get the optimal thresholds values.

Notwithstanding, these approaches have limitations; for example, they are computationally expensive, significantly when the number of thresholds is increased. Therefore, multilevel thresholding is considered a particular challenge that needs to be optimized. For these reasons, meta-heuristic methods are commonly utilized in the related literature to solve these problems [14].

MAs are inspired by nature, including areas such as physics, biology, and social behavior. Owing to their easy implementation, flexibility, and high performance, many scholars have used them to determine the optimal values for real-world problems [15,16,17,18,19,20]. Over the past years, many meta-heuristic algorithms have been proposed. For instance, Particle Swarm Optimization (PSO) [21], Differential Evolution (DE) [22], Genetic Algorithm [23], Teaching-Learning-based Optimization (TLBO) [24], Simulated Annealing (SA) [25], Gravity Search Algorithm (GSA) [26], and Ant Colony Optimization Algorithm (ACO) [27]. Other than these classic algorithms, many novel MAs have been proposed in the literature and widely used in different domains, such as Gray Wolf Optimization (GWO) [28], Whale Optimization Algorithm (WOA) [29], Salp Swarm Algorithm (SSA) [30], Sine Cosine Algorithm (SCA) [31], Arithmetic Optimization Algorithm (AOA) [32], Aquila Optimizer (AO) [33], Multi-Verse Optimization (MVO) [34], Slime Mould Algorithm (SMA) [35], and Remora Optimization Algorithm (ROA) [36].

In the literature, many works show the efficiency of MAs in obtaining optimal thresholds; the following are a few outstanding research works. Jia et al. [37] proposed an improved moth-flame optimization for color image segmentation using Otsu’s between-class variance and Kapur’s entropy as objective functions. The proposed method was compared with FPA, ACO, PSO, etc. Wu et al. [38] presented an ameliorated teaching-learning-based optimization based on a random learning method for multilevel thresholding using Kapur’s entropy and Otsu’s between-class variance. Pare et al. [39] proposed a color image multilevel segmentation strategy based on the Bat algorithm and Renyi’s entropy as the criterion to tackle the problems of multi-thresholding. Zhao et al. [40] presented a variant of SMA based on diffusion mechanism and association strategy for CT image segmentation. In this work, Renyi’s entropy was the objective fitness function. All of these works are examples of meta-heuristic algorithms applied in multilevel thresholding image segmentation. Generally, they provide good results on some benchmark images. However, considering the No Free Lunch (NFL) theorem proposed by Wolpert in 1997 [41], no unique optimization algorithm is available for solving all optimization problems. Furthermore, all meta-heuristic algorithms have limitations that affect the optimization capability, such as showing low convergence speed and unbalancing the exploration and exploitation ability.

Slime mould algorithm (SMA) is a novel meta-heuristic algorithm proposed by Li et al. in 2020 [35], which is inspired by the oscillation mode and behavior of slime mould in foraging. Since SMA has few parameters and shows better performance in specific fields, many scholars utilize it to solve questions of reality, such as parameter optimization of the fuzzy system and feature selection [36,37]. However, similar to other MAs, SMA may fall into local optimal and slow convergence speed in some optimization problems. Thus, many contributed works are proposed to enhance the performance of SMA. Dhawale et al. [42] suggested an improved SMA based on a chaotic strategy for solving global optimization and constrained engineering problems. Mostafa et al. [43] presented a modified SMA by adaptive weight to estimate the PV panel parameters. Hassan et al. [44] proposed an improved SMA via sine and cosine operators for solving economic and emission dispatch problems. Ewees et al. [45] integrated the SMA and firefly algorithm to improve the performance for feature selection.

While these proposed improved versions of the SMA algorithm are better than the original SMA algorithm on specific problems, when solving multilevel thresholding image segmentation, the imbalance between exploration and exploitation is still an unavoidable problem. This paper proposes a novel variant of SMA (ESMA) with the Levy flight and quasi opposition-based learning to tackle these shortcomings and obtain high-quality threshold values in image segmentation. The improvement involves two primary approaches. Firstly, the Levy flight strategy is applied to improve the exploration capability of SMA. Moreover, a novel variant of opposition-based learning (OBL), called quasi opposition-based learning (QOBL), is utilized to improve the ability to jump out the local optimal and balance the exploration and exploitation. In the experimental phase, the proposed ESMA is then tested on the 23 benchmark functions and applied to solve the multilevel thresholding image segmentation problem.

Meanwhile, the ESMA is also used to compare with other MAs. Furthermore, for the field of image segmentation, we evaluated the image segmentation results using Peak Signal to Noise Ratio (PSNR), Structural Similarity Index (SSIM), and Feature Similarity Index (FSIM). The experimental results illustrate that the proposed algorithm can produce high-quality results for benchmark functions and the image segmentation field.

Specifically, the main contributions of this paper can be summarized as follows:

- ESMA based on Levy flight and quasi opposition-based learning for solving global optimization problems and multilevel thresholding image segmentation.
- The optimization performance of ESMA is evaluated on 23 benchmark functions including unimodal and multimodal.
- DSMA is applied for thresholding segmentation using minimum cross-entropy measure.
- The segmentation quality is verified according to the PSNR, SSIM, FSIM, and statistical test.
- The performance of DSMA is compared with several classical and state-of-the-art optimization algorithm.

The remainder of this paper can be organized as follows: Section 2 describes a brief overview of SMA, Levy flight, quasi opposition-based learning, and maximum cross-entropy measure. Section 3 provides the details of the proposed algorithm. The experimental results are discussed and analyzed in detail in Section 4 and Section 5. Finally, the conclusion and future work are discussed in Section 6.

## 2. Preliminaries

This section presents the main inspiration and mathematical model of the slime mould algorithm (SMA). Next, the improvement strategy including Levy flight, and quasi opposition-based learning will be described. Finally, we will describe the minimum cross-entropy measure.

### 2.1. Slime Mould Algorithm

The slime mould algorithm (SMA) is a meta-heuristic optimization algorithm proposed recently by Li et al. [35], which is inspired by the oscillation behavior of slime mould in foraging. Slime mould achieves positive and negative feedback according to the quality of the food source. If the quality of the food source is high, the slime mould will use the region-limited search strategy. Meanwhile, if the food source is of low quality, the slime mould will leave this area and move to another food source in search space. Furthermore, SMA also has a slight chance of *z* to reinitialize the population in the search space.

Based on the above description, the updating process of slime mould can be expressed as in the following equation:

(1) $$\vec{X\left(t+1\right)}=\left\{\begin{array}{c} \begin{array}{c} r_{2}\times \left(UB-LB\right)+LB,\;r_{1}<z\\ \vec{X_{b}\left(t\right)}+\vec{vb}\times \left(\vec{W}\cdot \vec{X_{A}\left(t\right)}-\vec{X_{B}\left(t\right)}\right),\;r_{3}<p \end{array}\\ \vec{vc}\times \vec{X\left(t\right)},\;r_{3}\geq p \end{array}\right.$$

where *z* denotes the probability of slime mould reinitializing, which is 0.03; *r*_1_, *r*_2_, and *r*_3_ denote the random value in [0,1]; *LB* and *UB* represent the lower and upper bound of search space, respectively; *t* is the current iteration. $\vec{X_{b}\left(t\right)}$ represents global best solution; both $\vec{X_{A}\left(t\right)}$ and $\vec{X_{B}\left(t\right)}$ denote the random individual; $\vec{vb}$ ∈ [−*a*,*a*], and $\vec{vc}$ decreases linearly from one to zero. $\vec{W}$ represents the weight of slime mould.

The *p* can be calculated as follows:

(2) $$p=tanh\left|S\left(i\right)-DF\right|$$

where *i* ∈ 1,2, …, *N*, *S*(*i*) is the sequence representing the fitness of search agents. *DF* indicates the best fitness obtained by the slime mould.

$\vec{vb}$ can be calculated as follows:

(3) $$\vec{vb}=[-a,a]$$

(4) $$a=arctanh(-\left(\frac{t}{T}\right)+1)$$

where *T* represents the maximum iteration.

Note that the coefficient $\vec{W}$ is an essential parameter, which simulates the oscillation frequency of slime mould under different food sources. The $\vec{W}$ can be calculated as follows:

(5) $$\vec{W\left(SmellIndex\left(i\right)\right)}=\left\{\begin{array}{l} 1+r_{4}\times log\left(\frac{bF-S\left(i\right)}{bF-wF}+1\right),condition\\ 1-r_{4}\times log\left(\frac{bF-S\left(i\right)}{bF-wF}+1\right),others \end{array}\right.$$

(6) SmellIndex=sort(S)

where *r*_4_ is a random value in [0,1]; bF and *wF* represent the best fitness and worst fitness obtained currently, respectively; *condition* indicates the rank first half of the search agent of *S*(*i*). The pseudo-code of SMA is shown in Algorithm 1.
**Algorithm 1** Pseudo-code of SMAInitialize the positions of search agent;**While** current iteration < maximum iteration **do**  Check if any search agent goes beyond the search space and amend it;  Calculate the fitness of all slime mould;  **For** each search agent **do**   Update positions by Equation (1);  **End For**  *t* = *t* + 1;**End While****Return** the best solution;

### 2.2. Levy Flight

**Figure 1:**
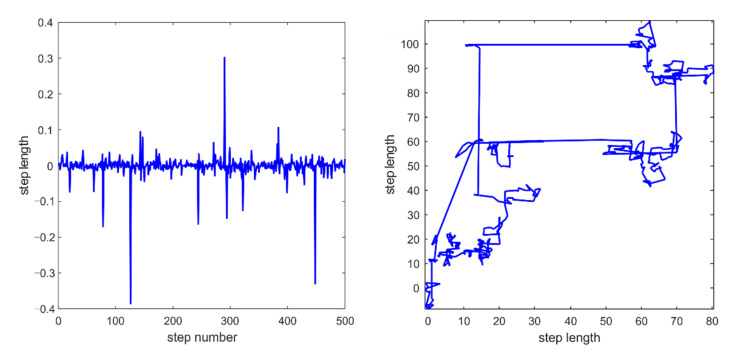
Levy distribution and 2D Levy trajectory.

Numerous studies reveal that the flight trajectories of many flying animals are consistent with characteristics typical of Levy flight. Levy flight is a class of non-Gaussian random walk that follows Levy distribution [46,47]. It performs occasional long-distance walking with frequent short-distance steps, as shown in Figure 1. The mathematical formula for Levy flight is as follows:

(7) $$Levy=0.01\times \frac{r_{5}\times \sigma }{{\left|r_{6}\right|}^{\frac{1}{\beta }}}$$

(8) $$\sigma ={\left(\frac{\Gamma \left(1+\beta \right)\times \sin\left(\frac{\pi \beta }{2}\right)}{\Gamma \left(\frac{1+\beta }{2}\right)\times \beta \times 2^{\left(\frac{\beta -1}{2}\right)}}\right)}^{\frac{1}{\beta }}$$

where *r*_4_ and *r*_5_ are random values in [0,1], and *β* is a constant equal to 1.5.

### 2.3. Quasi Opposition-Based Learning

#### 2.3.1. Opposition-Based Learning

Opposition-based learning (OBL) is an efficient search approach to avoid premature convergence, which was proposed by Tizhoosh in 2005 [48]. The main idea of OBL is to generate the opposite solution in the search space, then evaluate the original solution and its opposite solution by the objective function, respectively. Next, the best solution will be retained and go into the next iteration. Typically, the OBL strategy has high opportunities to provide closer optimal solutions than random ones.

We assume *x* to be an actual number in one dimension. Its opposite number *x_obl_* can be calculated by:

(9) $$x_{obl}=LB+UB-x$$

#### 2.3.2. Quasi Opposition-Based Learning

Based on the above description, a variant of OBL called quasi opposition-based learning (QOBL) was proposed by Rahnamayan et al. [49]. Unlike OBL, the QOBL strategy applied a quasi-opposite solution rather than the opposite solution. Therefore, the QOBL approach is more effective in finding globally optimal solutions than the previous strategy. On the basic theory of opposite solution, the quasi-opposite solution can be calculated by:

(10) $$x_{qobl}=rand(\frac{LB+UB}{2},x_{obl})$$

**Figure 2:**
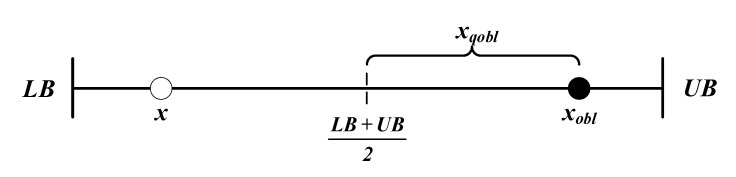
Diagram of OBL and QOBL.

To understand the above theory more clearly, Figure 2 illustrates the original solution *x*, its opposite solution *x_obl_*, and its quasi-opposite solution *x_qobl_*.

### 2.4. Minimum Cross-Entropy

In 1968, cross-entropy was proposed by Kullback [50]. Cross-entropy measures the difference information between two probability distributions $P=\left\{p_{1},\;p_{2},\;\ldots \;,\;p_{N}\right\}$ and $Q=\left\{q_{1},\;q_{2},\;\ldots \;,\;q_{N}\right\}$, defined by:

(11) $$D\left(P,\;Q\right)=\sum_{i=1}^{N}p_{i}\log\frac{p_{i}}{q_{i}}$$

In this work, we utilized minimum cross-entropy as a fitness function to find the optimal threshold value. The lower value of cross-entropy means less uncertainty and greater homogeneity. Let I be the origin grey image and *h*(*i*) be its histogram. Then, the thresholded image *I_th_* can be calculated as follows:

(12) $$I_{th}=\left\{\begin{array}{ll} \mu (1,\;th), & if\;I(x,y)<th\\ \mu (th,\;L+1), & if\;I(x,y)\geq th \end{array}\right.$$

where *th* denotes the threshold and divides the image into two different regions (foreground and background), and μ(a,b) can be calculated by:

(13) $$\mu (a,b)=\frac{\sum_{i=a}^{b-1}ih(i)}{\sum_{i=a}^{b-1}h(i)}$$

The cross-entropy can be computed by:

(14) $$f_{cross}(th)=\sum_{i=1}^{th-1}ih(i)\log\left(\frac{i}{\mu (1,\;th)}\right)+\sum_{i=th}^{L}ih(i)\log\left(\frac{i}{\mu (th,\;L+1)}\right)$$

The above objective functions are utilized to calculate the threshold value for bilevel thresholding. Thus it can be extended to a multilevel strategy. Yin [51] proposed a faster technique to obtain the threshold values for the digital image. The formula is as follows:

(15) $$f_{cross}(th)=\sum_{i=1}^{L}ih(i)\log i-\sum_{i=1}^{th-1}ih(i)\log(\mu (1,\;th))-\sum_{i=th}^{L}ih(i)\log(\mu (th,\;L+1))$$

where the above formula is based on thresholds $th=[th_{1},th_{2},\ldots ,th_{nt}]$, which contain *nt* different threshold values, by:

(16) $$f_{cross}(th)=\sum_{i=1}^{L}ih(i)\log(i)-\sum_{i=1}^{nt}H_{i}$$

where *nt* represents the total number of thresholds and *H_i_* can be defined as follows:

(17) $$H_{1}=\sum_{i=1}^{th_{1}-1}ih(i)\log(\mu (1,\;th_{1}))$$

(18) $$H_{k}=\sum_{i=th_{k-1}}^{th_{k}-1}ih(i)\log(\mu (th_{k-1},\;th_{k})),\;1<k<nt$$

(19) $$H_{nt}=\sum_{i=th_{nt}}^{L}ih(i)\log(\mu (th_{nt},\;L+1))$$

## 3. The Proposed Algorithm

### 3.1. Details of ESMA

The standard slime mould algorithm is a simple and efficient approach to solving specific optimization problems. However, based on the NFL theorem, no unique optimization algorithm is available for solving all optimization problems. Furthermore, SMA may be trapped into local optimal and show unperfected convergence speed for specific problems such as multilevel thresholding image segmentation. In order to improve the search ability and balance exploration and exploitation, in this paper, we propose an enhanced slime mould algorithm (ESMA) to improve the optimization performance. The improvement involves two major methods. Firstly, the Levy flight was used to enhance the exploration ability of SMA, which can be calculated by:

(20) $$\vec{X\left(t+1\right)}=\left\{\begin{array}{c} \begin{array}{c} r_{2}\times \left(UB-LB\right)+LB,\;r_{1}<z\\ X_{b}+vb\times \left(W\times X_{A}-X_{B}\right)\times Levy,\;r_{3}<p \end{array}\\ vc\times X_{i},\;r_{3}\geq p \end{array}\right.$$

**Figure 3:**
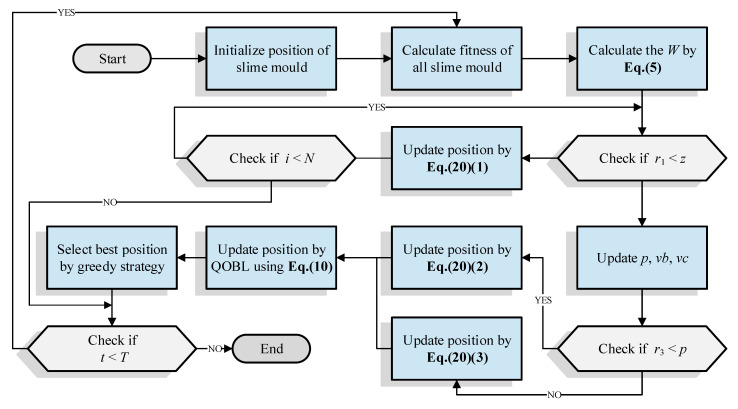
The flowchart of ESMA.

Secondly, quasi opposition-based learning was used to enhance the exploitation ability of SMA and balance the exploration and exploitation capability. The pseudo-code of ESMA is shown in Algorithm 2, and Figure 3 illustrates the flowchart of the proposed algorithm.
**Algorithm 2** Pseudo-code of ESMAInitialize the positions of search agent;**While** current iteration < maximum iteration **do**  Check if any search agent goes beyond the search space and amend it;  Calculate the fitness of all slime mould;  **For** each search agent, **do**   Update positions by Equation (20);  **End For**  Apply QOBL strategy by Equation (10);  Select the best position into next iteration by greedy strategy;  *t* = *t* + 1;**End While****Return** the best solution;

### 3.2. Computational Complexity Analysis

As can be seen, the ESMA mainly contains three components: Initialization phase, fitness evaluation, and position update procedure. In the initialization phase, the complexity can be expressed as O(*N*×*D*), where *N* represents the population size, and *D* denotes the dimension size of problems. Besides, the proposed algorithm evaluates the fitness of all slime mould with the complexity of O(*N*). The position update phase in the ESMA requires O(*N*×*D*). During the position updating phase, we utilize the QOBL to improve the exploitation ability and balance the exploration and exploitation; thus the QOBL strategy requires O(*N*×*D*). In summary, the total computation complexity of ESMA can be expressed as O(*N*×*D*×*T*) for *T* iterations. So, it can be concluded that both the SMA and ESMA have the same computational complexity wise.

## 4. Experimental Results and Discussion

### 4.1. Definition of 23 Benchmark Functions

To evaluate the exploration ability, exploitation ability, and escaping from the local optima ability of ESMA, twenty-three benchmark functions, including unimodal (F1–F7), multimodal (F8–F13), and fixed-dimension multimodal (F14–F23), are introduced [52]. The description of these functions is shown in Table 1, Table 2 and Table 3. As can be seen, the unimodal benchmark functions have only one global optimal value, which is suitable for evaluating the algorithms’ exploitation capability. Unlike unimodal functions, the multimodal and fixed-dimension benchmark functions have multiple local optimal values and only one optimal global value; it is suitable for evaluating the exploration ability and escaping from local minima.

To verify the performance of the proposed ESMA, we compared it with seven other algorithms including slime mould algorithm (SMA) [35], remora optimization algorithm (ROA) [36], arithmetic optimization algorithm (AOA) [32], aquila optimizer (AO) [33], salp swarm algorithm (SSA) [30], whale optimization algorithm (WOA) [29], and sine cosine algorithm (SCA) [31]. These classical and state-of-the-art algorithms are proved to equip with excellent performance on some optimization problems. The details of these algorithms are listed as follows:

- SMA [35] was proposed by Li et al. in 2020 and simulates the behavior and morphological process of slime mould during foraging.
- ROA [36] was proposed by Jia et al. in 2021 and simulates the parasitic behavior of remora.
- AOA [32] was proposed by Abualigah et al. in 2021 and is inspired by the arithmetic operator in mathematics.
- AO [33] was proposed by Abualigah et al. in 2021 and is inspired by the Aquila’s behaviors in nature during the process of catching the prey.
- SSA [30] was proposed by Mirjalili et al. in 2017 and is inspired by the swarming behavior of salps when navigating and foraging in oceans.
- WOA [29] was proposed by Mirjalili et al. in 2016 and mimics the social behavior of humpback whales.
- SCA [31] was proposed by Mirjalili et al. in 2016 and is inspired by the sine function and cosine function in nature.

Table 4 illustrates the parameter setting of each algorithm. For all the algorithms included in the comparison, we set the population size *N* = 30, dimension size *D* = 30, and maximum iteration *T* = 500; all the tests had 30 independent runs. Furthermore, we extract the average results, standard deviations, and statistical tests to evaluate the performance; the best results will be listed in bold font.

### 4.2. Statistical Results on 23 Benchmark Functions

The statistical results on 23 benchmark functions can be seen in Table 5. From this table, it can be clearly seen that the ESMA is superior to other algorithms in most benchmark functions. For unimodal benchmark functions (F1–F7), ESMA can obtain theoretical optimal for F1 and F3, while others algorithms cannot find the optimal solution. While ESMA cannot find the theoretical optimal for F4, F5, and F7, the convergence accuracy and robustness are better than other algorithms. In general, the exploitation ability of SMA is enhanced by applying the QOBL strategy. For the multimodal benchmark functions and fixed-dimension multimodal benchmark functions, ESMA also provides more competitive results than others. ESMA can obtain the theoretical optimal for F8, F9, F11, F14, F16, F17, F19, and F21–F23. For F10, F12, F13, and F15, ESMA gets the optimal global solution compared to others. Consequently, it can be concluded that ESMA always maintains high convergence accuracy and high robustness compared to other algorithms on such benchmark functions.

### 4.3. Wilcoxon Rank-Sum Test

In order to verify the non-incidentalness of the experimental results, this paper carried out the Wilcoxon rank-sum test (WRS). WRS is a nonparametric statistical test used to test the statistical performance between the proposed algorithm and comparison group on different benchmark functions [53]. WRS is based here on a 5% significant level, if the *p*-values obtained are less than 0.05, it indicates that there is a significant difference between them; otherwise, the difference is not obvious. The *p*-values obtained by algorithms are listed in Table 6. From this table, we can see that ESMA provides the statistically significant results compared with other algorithms.

### 4.4. Convergence Behavior Analysis

**Figure 4:**
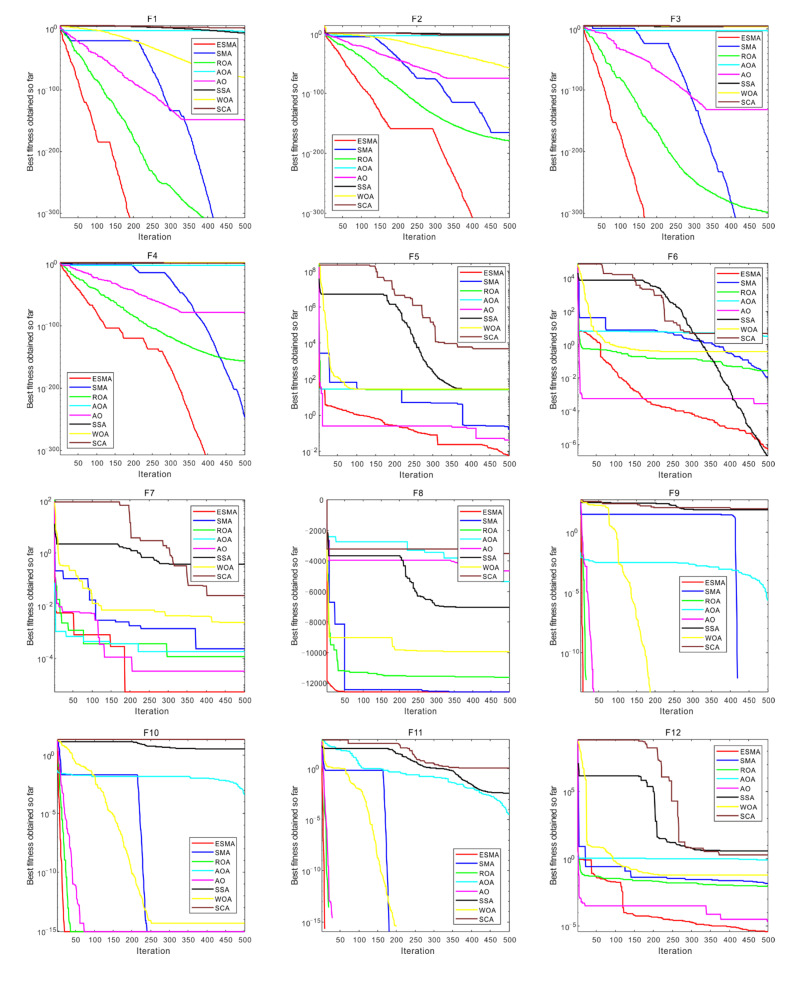

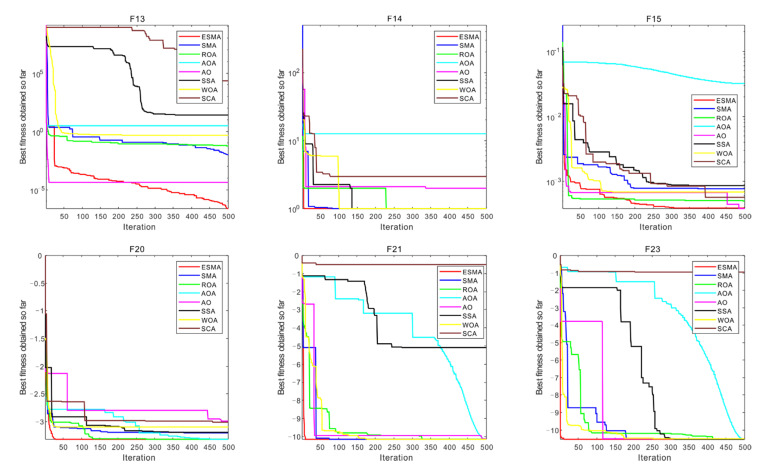
Convergence curve of algorithms obtained on 23 benchmark functions.

The convergence behavior of some benchmark functions is shown in Figure 4. On the unimodal benchmark functions, ESMA can achieve the highest accuracy and faster convergence speed. Especially for F1 and F3, while SMA can find the optimal solution, the convergence speed is slower than ESMA. For F2 and F4, ESMA finally converges to the optimal solution, while other algorithms either converge slowly or cannot converge to the optimal solution. For F5 and F7, while ESMA does not find the theoretical optimal solution, it still converges to the global optimal solution. On the multimodal benchmark functions, ESMA still shows the fastest convergence speed on most functions. While the global optimal solution is not found in some functions, it still has good performance compared with other algorithms. On the fixed dimensional multimodal functions, ESMA shows a faster convergence speed in the initial stage than others, and it also has a good convergence speed.

Generally, ESMA can obtain competitive results compared to other algorithms, such as the fastest convergence speed and highest convergence accuracy.

### 4.5. Qualitative Metrics Analysis

**Figure 5:**
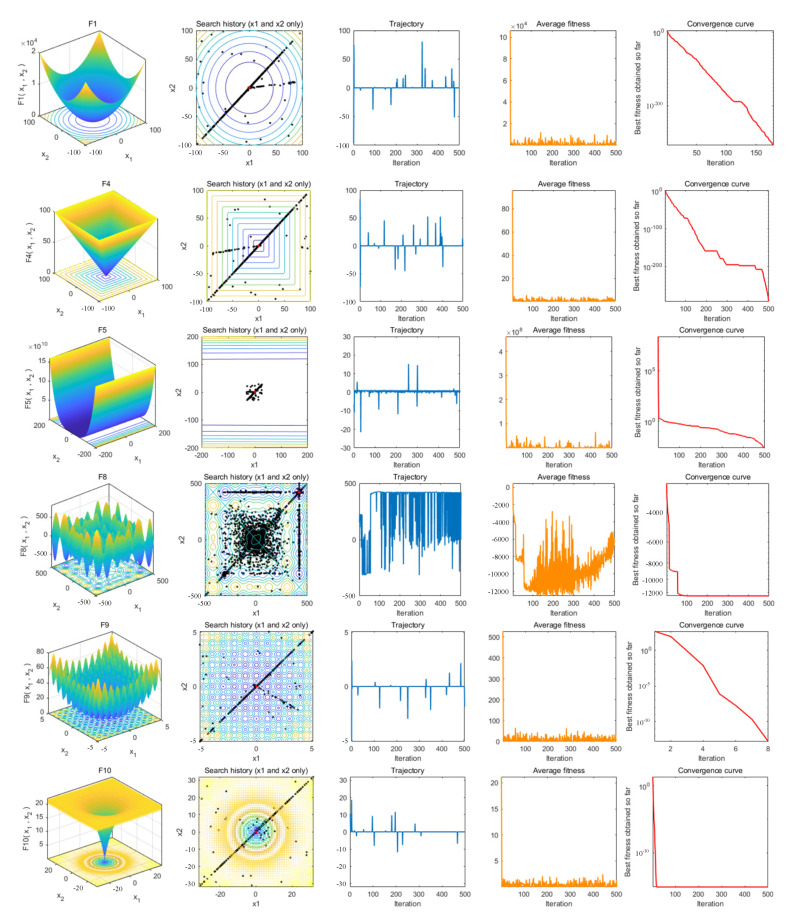

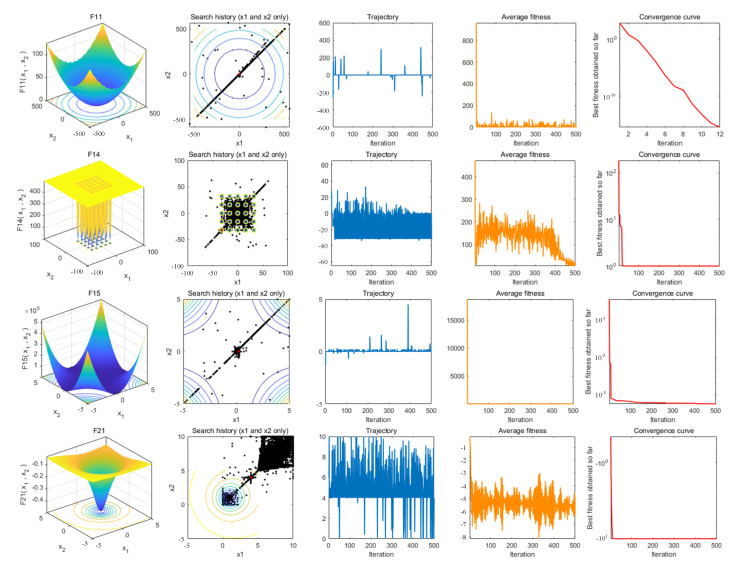
Qualitative metrics on some functions.

To evaluate the optimization performance of ESMA, Figure 5 illustrates the qualitative metrics, which include the 2D shape of benchmark functions (first column), search history of individuals (second column), trajectory (third column), average fitness (fourth column), and convergence curve (fifth column). For the first column, the 2D view of benchmark functions is described and shows the complexity of different functions. The second column illustrates the search history of the search agent from the first to the last iteration; it can be seen that the proposed ESMA is able to find the areas where the fitness values are the lowest. The trajectory of the first agent in the first dimension is described in the third column. We can see that the search agent oscillates continuously in the search space, which shows that the search agent widely studies the most promising fields and better solutions. The fourth column denotes the average fitness history. It can be seen that the fitness curve is decreasing, which indicates that the quality of the population is improving at each iteration. The last column is the convergence curve, which reveals that populations find the best solution after each iteration.

## 5. Experimental Results on Multilevel Thresholding

This section introduces the experimental details of the proposed algorithm ESMA applied to the multilevel thresholding image segmentation. First, the benchmark images and the experimental setup are presented in Section 5.1. Furthermore, the results of the algorithms in fitness, PSNR, SSIM, and FSIM are also analyzed. This section also shows the statistical analysis used to compare the proposed algorithm with other competitive algorithms.

### 5.1. Experiment Setup

**Figure 6:**
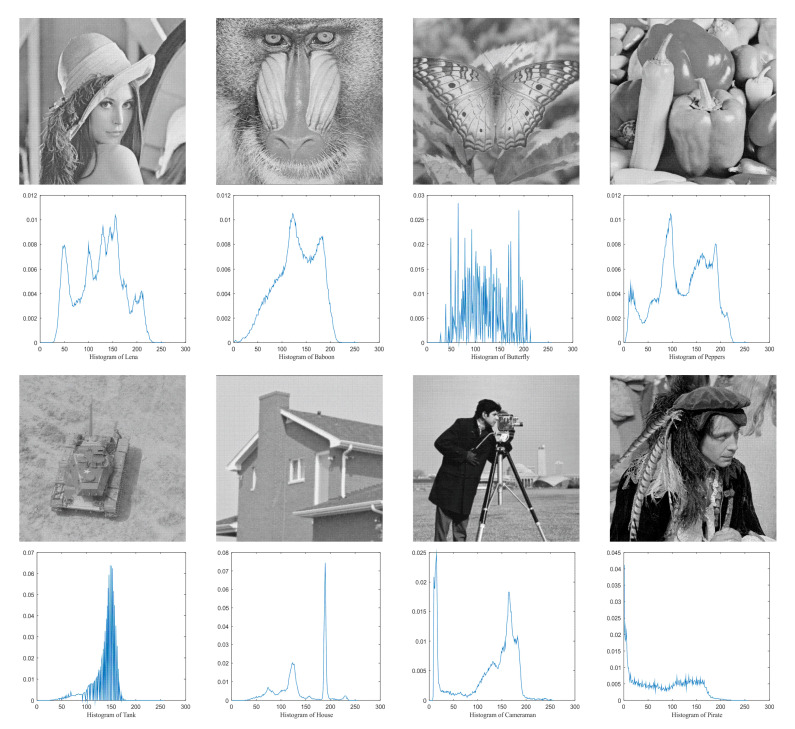
Benchmark images.

In this paper, the benchmark greyscale images, including Lena, Baboon, Butterfly, etc., are used to evaluate the performance of the proposed algorithm ESMA’s image segmentation [54]. All the benchmark images and their histogram images are represented in Figure 6. To guarantee the fairness of the experiment, all the algorithms are evaluated 30 times per image, and the maximum iteration *T* is 500; the number of population size *N* is 30. The number of thresholds values [nTh = 4, 6, 8, 10].

### 5.2. Evaluation Measurements

In this paper, three common evaluation methods are used to illustrate the performance of the algorithm and the quality of image segmentation, namely PSNR, FSIM, and SSIM, which are defined as follows:

#### 5.2.1. PSNR

Peak Signal to Noise Ratio (PSNR) is an image quality evaluation metric used to evaluate the similarity between the original image and the segmented image [55]. The PSNR is calculated as:

(21) $$PSNR=20log_{10}\frac{255}{RMSE}$$

(22) $$RMSE=\sqrt{\frac{\sum_{i=1}^{M}\sum_{j=1}^{N}\left({\left(I(i,j)-Seg(i,j)\right)}^{2}\right)}{M\times N}}$$

where *I* and *Seg* denote the original image and segmented image with *M* × *N*, respectively; RMSE is the root mean square error.

#### 5.2.2. SSIM

Structural Similarity (SSIM) is a common metric used to measure the structural similarity between the original image and the segmented image [3], and is defined as:

(23) $$SSIM(I,Seg)=\frac{\left(2\mu_{I}\mu_{Seg}+c_{1})(2\sigma_{I,Seg}+c_{2}\right)}{\left({\mu_{I}}^{2}+{\mu_{Seg}}^{2}+c_{1})({\sigma_{I}}^{2}+{\sigma_{Seg}}^{2}+c_{2}\right)}$$

where *μ_I_* and *μ_Seg_* indicate the mean intensity of the original image and its segmented image; *σ_I_* and *σ_Seg_* denote the standard deviation of the original image and its segmented image; *σ_I,Seg_* is the covariance of the original image and the segmented image. *c*_1_ and *c*_2_ are constant.

#### 5.2.3. FSIM

Feature Similarity (FSIM) is used to estimate the structural similarity between the original image and the segmented image [56], and is defined as:

(24) $$FSIM=\frac{\sum_{\omega \in \Omega }S_{L}(\omega )PC_{m}(\omega )}{\sum_{\omega \in \Omega }PC_{m}(\omega )}$$

(25) $$S_{L}(\omega )=S_{PC}(\omega )S_{G}(\omega )$$

(26) $$S_{PC}(\omega )=\frac{2PC_{1}(\omega )PC_{2}(\omega )+T_{1}}{P{C_{1}}^{2}(\omega )+PC_{2}{}^{2}(\omega )+T_{1}}$$

(27) $$S_{G}(\omega )=\frac{2G_{1}(\omega )G_{2}(\omega )+T_{2}}{{G_{1}}^{2}(\omega )+{G_{2}}^{2}(\omega )+T_{2}}$$

where Ω indicates the entire image domain; *PC*_1_ and *PC*_2_ represent the phase consistency of the original image and its segmented image, respectively; *G*_1_ and *G*_2_ represent the gradient magnitude of the original image and segmented image, respectively. *T*_1_ and *T*_2_ both are constant.

### 5.3. Experimental Result Analysis

This section mainly compares ESMA with seven optimization algorithms: SMA, ROA, AOA, AO, SSA, WOA, and SCA. All the algorithms run independently 30 times, and the average value (mean) and standard deviation (Std) are selected as the evaluation indexes, in which the best values are marked in bold.

Table A1 illustrates the optimal threshold values obtained by different algorithms on the benchmark images. It can be seen that when the number of thresholds is equal to 4 and 6, the thresholds obtained by most algorithms are roughly the same. However, the results are quite different when the thresholds are extended to 8 and 10, especially for SCA and AOA.

Table A2 represents the average fitness values and their Std obtained by all algorithms on the benchmark images. In general, the lower value of the average fitness denotes the better quality of segmentation. It can be seen that the fitness value of ESMA is better than most algorithms. For example, when the tank image is segmented with ten threshold levels, the fitness value obtained by ESMA ranks first, which is greatly improved compared with the SMA. Experimental results show that ESMA has better performance and strong applicability in segmenting multilevel threshold images.

Table A3 shows the PSNR results obtained by all algorithms. As mentioned above, it is suitable to evaluate the similarity between the segmented image and the original image, where a higher average value indicates a better segmentation quality. From the attained results, however, there are only small differences between the ESMA and other compared algorithms in threshold values 4 and 6. However, the PSNR values significantly increase when the threshold values are increasing. It can be observed that, for most benchmark images, the proposed ESMA significantly produces more favorable and reliable results than the original SMA and other compared algorithms, which provides better PSNR results for most benchmark images, for example, when images Lena, Baboon, Tank, Cameraman, and Pirate are tackled with 10 threshold levels. Obviously, the PSNR values are highest, and AO and WOA are ranked second and third, respectively. When segmenting Lena and Baboon images, ESMA showed the best PSNR value among all thresholds. Generally, ESMA presents the best performance with the images Lena, Baboon, Peppers, Tank, and House.

Table A4 illustrates the SSIM value obtained from different algorithms. As is possible to obverse, when the threshold is equal to 4, the SSIM results of each algorithm are roughly the same. Then, as the number of threshold values increases, the value of SSIM continues to increase, ESMA can obtain more original image information than other algorithms. For example, when the threshold value is equal to 4, the SSIM value obtained by ESMA for Baboon is 0.8041. When the number of thresholds increases to 10, the SSIM is 0.9395. Furthermore, when the threshold is equal to 6, 8, and 10, the segmentation quality of ESMA is better than most comparison algorithms, especially for segmenting Baboon, Butterfly, and House. In the case of Cameraman, the best SSIM results were obtained by ROA in the threshold values 4, 6, and 8. Overall, ESMA ranked first in segmentation quality.

Table A5 shows the FSIM values obtained by different algorithms, where a higher value represents the best quality of the segmentation. We can see that the SMA and ROA show significant performance in Baboon, Butterfly, and Cameraman. Both AOA and SCA are not shown a significant performance for any of the images. The proposed ESMA can achieve good results in segmenting most images. For example, when the House image is processed using eight each threshold level, the value of FSIM is significant. Therefore, in most cases, the algorithm proposed in this paper can extract the interesting target from the image more accurately.

Table A6 represents the *p*-value obtained by Wilcoxon rank-sum test with 5% significance level. It can be seen from the results that ESMA is significantly different from ROA, AOA, SSA, and SCA, which means that the proposed algorithm ESMA has been improved considerably. However, there is no significant difference at Lena for level 4. When comparing ESMA and WOA, there are significant differences in other images except for Butterfly, House, and Pepper.

Table 7 shows the image segmentation results of the proposed algorithm ESMA for different thresholds, in which the obtained optimal threshold is marked with a red vertical line. This table shows how the thresholds divide an image into several different classes and how the objects are segmented from the background.

**Figure 7:**
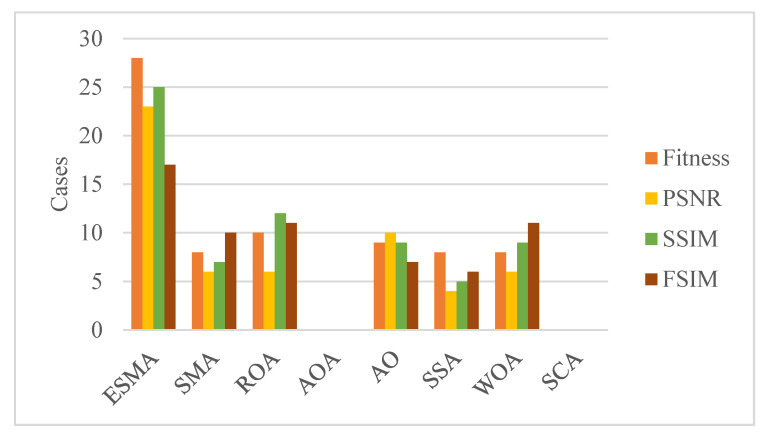
Summary of Fitness, PSNR, SSIM, and FSIM number of best cases for all thresholds obtained by algorithms.

Figure 7 summarizes the segmentation experimental results of fitness, PSNR, SSIM, and FSIM based on the objective function. From this figure, we can see that the segmentation performance of ESMA is significantly improved compared with original SMA, and ROA and WOA are ranked second and third, respectively.

According to the above evaluation metrics and statistical test, the proposed ESMA has a better segmentation quality than other compared algorithms. Thus, the proposed ESMA can be effectively applied to the field of image segmentation.

## 6. Conclusions and Future Work

In this paper, an enhanced slime mould algorithm (ESMA) is proposed for global optimization and multilevel thresholding image segmentation. In order to improve the performance of SMA, we use two strategies. First, the Levy flight strategy is used to enhance the exploration ability. Second, quasi opposition-based learning is used to enhance the exploitation ability and balance the exploration and exploitation. To evaluate the performance of ESMA, ESMA and some state-of-the-art algorithms were tested on the 23 benchmark functions, and the results indicate that the ESMA is superior to others. This shows that the above two strategies can effectively help SMA avoid falling into optimal local state and improve the global search ability of the population. In addition, we applied ESMA to multilevel thresholding image segmentation, and minimum cross-entropy is selected as the fitness function. The experimental evaluation metrics determined the mean fitness, standard deviation, PSNR, SSIM, FSIM and Wilcoxon rank-sum test. Experimental results show that the ESMA method is superior to other image segmentation methods in PSNR, FSIM, SSIM, and statistical tests.

While the proposed work is valuable in the image segmentation field, it is necessary to extend the benchmark images and increase the number of thresholds to obtain more reliable results. In addition, we will also seek to hybridize the ESMA with other MAs to improve the segmentation results when solving real-world applications, such as ship target segmentation and medical image segmentation. Meanwhile, other objective functions can be selected to realize multilevel thresholding image segmentation.

## Figures and Tables

**Table 1:** Unimodal benchmark functions.

Function	Dim	Range	*f_min_*
$F_{1}(x)=\sum_{i=1}^{n}x_{i}^{2}$	30	[−100,100]	0
$F_{2}(x)=\sum_{i=1}^{n}\left|x_{i}\right|+\prod_{i=1}^{n}\left|x_{i}\right|$	30	[−10,10]	0
$F_{3}(x)={\sum_{i=1}^{n}\left(\sum_{j-1}^{i}x_{j}\right)}^{2}$	30	[−100,100]	0
$F_{4}(x)=\max_{i}\{\left|x_{i}\right|,1\leq i\leq n\}$	30	[−100,100]	0
$F_{5}(x)=\sum_{i=1}^{n-1}\left[100{\left(x_{i+1}-x_{i}^{2}\right)}^{2}+{\left(x_{i}-1\right)}^{2}\right]$	30	[−30,30]	0
$F_{6}(x)=\sum_{i=1}^{n}{\left(x_{i}+5\right)}^{2}$	30	[−100,100]	0
$F_{7}(x)=\sum_{i=1}^{n}ix_{i}^{4}+random[0,1)$	30	[−1.28,1.28]	0

**Table 2:** Multimodal benchmark functions.

Function	Dim	Range	*f_min_*
$F_{8}(x)=\sum_{i=1}^{n}-x_{i}\sin(\sqrt{\left|x_{i}\right|})$	30	[−500,500]	−12,569.487
$F_{9}(x)=\sum_{i=1}^{n}\left[x_{i}^{2}-10\cos(2\pi x_{i})+10\right]$	30	[−5.12,5.12]	0
$F_{10}(x)=-20\exp(-0.2\sqrt{\frac{1}{n}\sum_{i=1}^{n}x_{i}^{2}})-\exp(\frac{1}{n}\sum_{i=1}^{n}\cos(2\pi x_{i}))+20+e$	30	[−32,32]	0
$F_{11}(x)=\frac{1}{4000}\sum_{i=1}^{n}x_{i}^{2}-\prod_{i=1}^{n}\cos(\frac{x_{i}}{\sqrt{i}})+1$	30	[−600,600]	0
$\begin{array}{l} F_{12}(x)=\frac{\pi }{n}\{10\sin(\pi y_{1})+{\sum_{i=1}^{n-1}\left(y_{i}-1\right)}^{2}[1+10\sin^{2}(\pi y_{i+1})]+{\left(y_{n}-1\right)}^{2}\}\\ +\sum_{i=1}^{n}u(x_{i},10,100,4),\mathrm{where}\;y_{i}=1+\frac{x_{i}+1}{4},\\ u(x_{i},a,k,m)=\left\{\begin{array}{cc} k{\left(x_{i}-a\right)}^{m} & x_{i}>a\\ 0 & -a<x_{i}<a\\ k{\left(-x_{i}-a\right)}^{m} & x_{i}<-a \end{array}\right. \end{array}$	30	[−50,50]	0
$\begin{array}{l} F_{13}(x)=0.1(\sin^{2}(3\pi x_{1})+\sum_{i=1}^{n}{\left(x_{i}-1\right)}^{2}[1+\sin^{2}(3\pi x_{i}+1)]\\ +{\left(x_{n}-1\right)}^{2}[1+\sin^{2}(2\pi x_{n})])+\sum_{i=1}^{n}u(x_{i},5,100,4) \end{array}$	30	[−50,50]	0

**Table 3:** Fixed-dimension multimodal benchmark functions.

Function	Dim	Range	*f_min_*
$F_{14}(x)={\left(\frac{1}{500}+\sum_{j=1}^{25}\frac{1}{j+\sum_{i=1}^{2}{\left(x_{i}-a_{ij}\right)}^{6}}\right)}^{-1}$	2	[−65,65]	0.998
$F_{15}(x)=\sum_{i=1}^{11}{\left[a_{i}-\frac{x_{1}(b_{i}^{2}+b_{i}x_{2})}{b_{i}^{2}+b_{i}x_{3}+x_{4}}\right]}^{2}$	4	[−5,5]	0.00030
$F_{16}(x)=4x_{1}^{2}-2.1x_{1}^{4}+\frac{1}{3}x_{1}^{6}+x_{1}x_{2}-4x_{2}^{2}+x_{2}^{4}$	2	[−5,5]	−1.0316
$F_{17}(x)={\left(x_{2}-\frac{5.1}{4\pi^{2}}x_{1}^{2}+\frac{5}{\pi }x_{1}-6\right)}^{2}+10(1-\frac{1}{8\pi })\cos x_{1}+10$	2	[−5,5]	0.398
$\begin{array}{l} F_{18}(x)=[1+{\left(x_{1}+x_{2}+1\right)}^{2}(19-14x_{1}+3x_{1}^{2}-14x_{2}+6x_{1}x_{2}+3x_{2}^{2})]\\ \times [30+{\left(2x_{1}-3x_{2}\right)}^{2}\times (18-32x_{2}+12x_{1}^{2}+48x_{2}-36x_{1}x_{2}+27x_{2}^{2})] \end{array}$	2	[−2,2]	3
$F_{19}(x)=-\sum_{i=1}^{4}c_{i}\exp(-\sum_{j=1}^{3}a_{ij}{\left(x_{j}-p_{ij}\right)}^{2})$	3	[−1,2]	−3.86
$F_{20}(x)=-\sum_{i=1}^{4}c_{i}\exp(-\sum_{j=1}^{6}a_{ij}{\left(x_{j}-p_{ij}\right)}^{2})$	6	[0,1]	−3.32
$F_{21}(x)=-{\sum_{i=1}^{5}\left[(X-a_{i}){\left(X-a_{i}\right)}^{T}+c_{i}\right]}^{-1}$	4	[0,10]	−10.1532
$F_{22}(x)=-{\sum_{i=1}^{7}\left[(X-a_{i}){\left(X-a_{i}\right)}^{T}+c_{i}\right]}^{-1}$	4	[0,10]	−10.4028
$F_{23}(x)=-{\sum_{i=1}^{10}\left[(X-a_{i}){\left(X-a_{i}\right)}^{T}+c_{i}\right]}^{-1}$	4	[0,10]	−10.5363

**Table 4:** Parameter settings for the comparative algorithms.

Algorithm	Parameters
SMA [35]	*z* = 0.03
ROA [36]	*c* = 0.1
AOA [32]	*α* = 5; *μ* = 0.5;
AO [33]	*U* = 0.00565; *c* = 10; *ω* = 0.005; *α* = 0.1; *δ* = 0.1;
SSA [30]	*c*_1_ = [1,0]; *c*_2_∈[0,1]; *c*_3_∈[0,1]
WOA [29]	*a*_1_ = [2,0]; *a*_2_ = [−1,−2]; *b* = 1
SCA [31]	*a* = [2,0]

**Table 5:** Simulation results for 23 benchmark functions.

Function		ESMA	SMA	ROA	AOA	AO	SSA	WOA	SCA
F1	Mean	**0.00 × 10^+00^**	3.83 × 10^−320^	5.93× 10^−323^	2.05× 10^−13^	1.19 × 10^−104^	1.31 × 10^−07^	2.30 × 10^−68^	2.25 × 10^+01^
	Std	**0.00 × 10^+00^**	**0.00 × 10^00^**	**0.00 × 10^00^**	1.12 × 10^−12^	6.49 × 10^−104^	1.15 × 10^−07^	1.26 × 10^−67^	6.73 × 10^+01^
F2	Mean	1.12 × 10^−188^	1.68 × 10^−148^	6.68 × 10^−162^	**0.00 × 10^+00^**	2.45 × 10^−53^	1.96 × 10^+00^	3.57 × 10^−52^	1.84 × 10^−02^
	Std	0.00 × 10^+00^	9.20 × 10^−148^	3.61 × 10^−161^	**0.00 × 10^+00^**	1.34 × 10^−52^	1.49 × 10^+00^	8.24 × 10^−52^	3.52 × 10^−02^
F3	Mean	**0.00 × 10^+00^**	3.03 × 10^−285^	5.68 × 10^−286^	3.47 × 10^−03^	3.16 × 10^−97^	1.66 × 10^+03^	4.50 × 10^+04^	1.04 × 10^+04^
	Std	**0.00 × 10^+00^**	**0.00 × 10^+00^**	**0.00 × 10^+00^**	8.24 × 10^−03^	1.73 × 10^−96^	1.32 × 10^+03^	1.64 × 10^+04^	5.62 × 10^+03^
F4	Mean	**5.48 × 10^−222^**	9.79 × 10^−161^	2.33 × 10^−153^	2.62 × 10^−02^	3.78 × 10^−53^	1.13 × 10^+01^	5.27 × 10^+01^	3.50 × 10^+01^
	Std	**0.00 × 10^+00^**	5.08 × 10^−160^	1.27 × 10^−152^	2.02 × 10^−02^	2.07 × 10^−52^	2.92 × 10^+00^	2.75 × 10^+01^	1.48 × 10^+01^
F5	Mean	**3.79 × 10^−03^**	6.04 × 10^+00^	2.71 × 10^+01^	2.83 × 10^+01^	4.02 × 10^−03^	1.78 × 10^+02^	2.79 × 10^+01^	9.83 × 10^+04^
	Std	**2.33 × 10^−03^**	1.01 × 10^+01^	4.41 × 10^−01^	4.22 × 10^−01^	7.30 × 10^−03^	3.08 × 10^+02^	4.92 × 10^−01^	1.99 × 10^+05^
F6	Mean	5.80 × 10^−07^	6.08 × 10^−03^	9.77 × 10^−02^	3.08 × 10^+00^	9.27 × 10^−05^	**1.71 × 10^−07^**	3.71 × 10^−01^	1.26 × 10^+01^
	Std	1.76 × 10^−07^	3.84 × 10^−03^	1.04 × 10^−01^	3.20 × 10^−01^	1.26 × 10^−04^	**1.50 × 10^−07^**	2.29 × 10^−01^	1.02 × 10^+01^
F7	Mean	**5.24 × 10^−05^**	1.84 × 10^−04^	1.48 × 10^−04^	5.37 × 10^−05^	7.57 × 10^−05^	1.61 × 10^−01^	4.74 × 10^−03^	9.19 × 10^−02^
	Std	**4.96 × 10^−05^**	1.50 × 10^−04^	1.27 × 10^−04^	4.21 × 10^−05^	7.75 × 10^−05^	7.12 × 10^−02^	6.51 × 10^−03^	1.01 × 10^−01^
F8	Mean	**−1.26 × 10^+04^**	−1.26 × 10^+04^	−1.24 × 10^+04^	−5.20 × 10^+03^	−8.88 × 10^+03^	−7.34 × 10^+03^	−1.03 × 10^+04^	−3.72 × 10^+03^
	Std	**4.07 × 10^−03^**	3.91 × 10^−01^	4.39 × 10^+02^	4.69 × 10^+02^	3.74 × 10^+03^	6.61 × 10^+02^	2.01 × 10^+03^	2.65 × 10^+02^
F9	Mean	**0.00 × 10^+00^**	**0.00 × 10^+00^**	**0.00 × 10^+00^**	**0.00 × 10^+00^**	**0.00 × 10^+00^**	5.79 × 10^+01^	4.11 × 10^+00^	4.28 × 10^+01^
	Std	**0.00 × 10^+00^**	**0.00 × 10^+00^**	**0.00 × 10^+00^**	**0.00 × 10^+00^**	**0.00 × 10^+00^**	1.87 × 10^+01^	2.25 × 10^+01^	3.24 × 10^+01^
F10	Mean	**8.88 × 10^−16^**	**8.88 × 10^−16^**	**8.88 × 10^−16^**	**8.88 × 10^−16^**	**8.88 × 10^−16^**	2.77 × 10^+00^	4.80 × 10^−15^	1.26 × 10^+01^
	Std	**0.00 × 10^+00^**	**0.00 × 10^+00^**	**0.00 × 10^+00^**	**0.00 × 10^+00^**	**0.00 × 10^+00^**	8.52 × 10^−01^	2.35 × 10^−15^	8.96 × 10^+00^
F11	Mean	**0.00 × 10^+00^**	**0.00 × 10^+00^**	**0.00 × 10^+00^**	**0.00 × 10^+00^**	**0.00 × 10^+00^**	1.78 × 10^−02^	0.00 × 10^+00^	9.69 × 10^−01^
	Std	**0.00 × 10^+00^**	**0.00 × 10^+00^**	**0.00 × 10^+00^**	**0.00 × 10^+00^**	**0.00 × 10^+00^**	1.23 × 10^−02^	0.00 × 10^+00^	3.69 × 10^−01^
F12	Mean	2.18 × 10^−05^	4.44 × 10^−03^	1.04 × 10^−02^	4.99 × 10^−01^	**2.64 × 10^−06^**	6.84 × 10^+00^	2.53 × 10^−02^	2.92 × 10^+05^
	Std	7.96 × 10^−05^	7.53 × 10^−03^	5.91 × 10^−03^	4.80 × 10^−02^	**5.61 × 10^−06^**	3.30 × 10^+00^	1.62 × 10^−02^	1.19 × 10^+06^
F13	Mean	**3.62 × 10^−07^**	5.78 × 10^−03^	2.25 × 10^−01^	2.83 × 10^+00^	1.99 × 10^−05^	1.56 × 10^+01^	5.31 × 10^−01^	4.50 × 10^+04^
	Std	**1.69 × 10^−07^**	5.70 × 10^−03^	1.51 × 10^−01^	1.08 × 10^−01^	3.79 × 10^−05^	1.47 × 10^+01^	2.84 × 10^−01^	1.76 × 10^+05^
F14	Mean	**9.98 × 10^−01^**	**9.98 × 10^−01^**	4.45 × 10^+00^	9.54 × 10^+00^	2.50 × 10^+00^	1.10 × 10^+00^	2.12 × 10^+00^	2.25 × 10^+00^
	Std	**5.17 × 10^−16^**	6.55 × 10^−13^	4.85 × 10^+00^	4.22 × 10^+00^	3.33 × 10^+00^	4.00 × 10^−01^	2.12 × 10^+00^	2.49 × 10^+00^
F15	Mean	6.07 × 10^−04^	5.57 × 10^−04^	**4.23 × 10^−04^**	1.80 × 10^−02^	4.89 × 10^−04^	2.92 × 10^−03^	5.83 × 10^−04^	8.49 × 10^−04^
	Std	2.67 × 10^−04^	2.83 × 10^−04^	2.92 × 10^−04^	2.86 × 10^−02^	3.29 × 10^−04^	5.93 × 10^−03^	3.84 × 10^−04^	**2.32 × 10^−04^**
F16	Mean	**−1.03 × 10^+00^**	**−1.03 × 10^+00^**	**−1.03 × 10^+00^**	**−1.03 × 10^+00^**	**−1.03 × 10^+00^**	**−1.03 × 10^+00^**	**−1.03 × 10^+00^**	**−1.03 × 10^+00^**
	Std	**7.70 × 10^−15^**	3.95 × 10^−10^	5.90 × 10^−08^	1.65 × 10^−07^	3.69 × 10^−04^	4.13 × 10^−14^	1.32 × 10^−09^	4.90 × 10^−05^
F17	Mean	**3.98 × 10^−01^**	**3.98 × 10^−01^**	**3.98 × 10^−01^**	**3.98 × 10^−01^**	**3.98 × 10^−01^**	**3.98 × 10^−01^**	**3.98 × 10^−01^**	4.00 × 10^−01^
	Std	**2.82 × 10^−13^**	2.77 × 10^−08^	4.26 × 10^−06^	8.49 × 10^−08^	2.67 × 10^−04^	9.08 × 10^−15^	5.79 × 10^−06^	2.15 × 10^−03^
F18	Mean	1.02 × 10^+01^	**3.00 × 10^+00^**	**3.00 × 10^+00^**	1.02 × 10^+01^	3.03 × 10^+00^	**3.00 × 10^+00^**	**3.00 × 10^+01^**	**3.00 × 10^+00^**
	Std	1.21 × 10^+01^	**7.33 × 10^−11^**	6.72 × 10^−05^	1.21 × 10^+01^	2.65 × 10^−02^	1.90 × 10^−13^	4.08 × 10^−05^	2.37 × 10^−04^
F19	Mean	**−3.86 × 10^+00^**	**−3.86 × 10^+00^**	**−3.86 × 10^+00^**	−3.85 × 10^+00^	−3.85 × 10^+00^	**−3.86 × 10^+00^**	−3.83 × 10^+00^	−3.85 × 10^+00^
	Std	**1.85 × 10^−11^**	5.00 × 10^−07^	2.07 × 10^−03^	6.68 × 10^−03^	9.15 × 10^−03^	6.05 × 10^−10^	1.40 × 10^−01^	1.17 × 10^−02^
F20	Mean	**−3.26 × 10^+00^**	−3.25 × 10^+00^	−3.28 × 10^+00^	−3.06 × 10^+00^	−3.17 × 10^+00^	−3.23 × 10^+00^	−3.18 × 10^+00^	−2.86 × 10^+00^
	Std	**3.05 × 10^−02^**	5.96 × 10^−02^	6.88 × 10^−02^	9.11 × 10^−02^	7.18 × 10^−02^	5.77 × 10^−02^	1.88 × 10^−01^	4.10 × 10^−01^
F21	Mean	**−1.02 × 10^+01^**	**−1.02 × 10^+01^**	−1.01 × 10^+01^	−3.47 × 10^+00^	−1.01 × 10^+01^	−7.73 × 10^+00^	−8.03 × 10^+00^	−2.73 × 10^+00^
	Std	**5.52 × 10^−08^**	3.30 × 10^−04^	1.25 × 10^−02^	1.24 × 10^+00^	3.68 × 10^−02^	3.32 × 10^+00^	2.89 × 10^+00^	2.28 × 10^+00^
F22	Mean	**−1.04 × 10^+01^**	**−1.04 × 10^+01^**	**−1.04 × 10^+01^**	−4.00 × 10^+00^	**−1.04 × 10^+01^**	−8.42 × 10^+00^	−7.67 × 10^+00^	−2.86 × 10^+00^
	Std	**5.77 × 10^−08^**	3.07 × 10^−04^	1.58 × 10^−02^	1.51 × 10^+00^	9.40 × 10^−03^	3.14 × 10^+00^	3.54 × 10^+00^	1.77 × 10^+00^
F23	Mean	**−1.05 × 10^+01^**	**−1.05 × 10^+01^**	**−1.05 × 10^+01^**	−3.97 × 10^+00^	**−1.05 × 10^+01^**	−8.00 × 10^+00^	−6.60 × 10^+00^	−3.31 × 10^+00^
	Std	**3.17 × 10^−08^**	3.92 × 10^−04^	1.94 × 10^−02^	1.63 × 10^+00^	2.59 × 10^−02^	3.47 × 10^+00^	3.32 × 10^+00^	1.98 × 10^+00^

**Table 6:** The results of the Wilcoxon rank-sum test were obtained by algorithms on 23 benchmark functions.

Function	ESMA vs.						
	SMA	ROA	AOA	AO	SSA	WOA	SCA
F1	3.51 × 10^−01^	3.97 × 10^−02^	6.87 × 10^−07^	6.87 × 10^−07^	6.87 × 10^−07^	6.87 × 10^−07^	6.87 × 10^−07^
F2	2.33 × 10^−05^	3.39 × 10^−06^	3.39 × 10^−06^	3.39 × 10^−06^	3.39 × 10^−06^	3.39 × 10^−06^	3.39 × 10^−06^
F3	1.64 × 10^−01^	6.87 × 10^−07^	6.87 × 10^−07^	6.87 × 10^−07^	6.87 × 10^−07^	6.87 × 10^−07^	6.87 × 10^−07^
F4	1.92 × 10^−05^	3.36 × 10^−06^	3.36 × 10^−06^	3.36 × 10^−06^	3.36 × 10^−06^	3.36 × 10^−06^	3.36 × 10^−06^
F5	3.39 × 10^−06^	3.39 × 10^−06^	3.39 × 10^−06^	2.15 × 10^−03^	3.39 × 10^−06^	3.39 × 10^−06^	3.39 × 10^−06^
F6	3.39 × 10^−06^	3.39 × 10^−06^	3.39 × 10^−06^	3.39 × 10^−06^	2.23 × 10^−04^	3.39 × 10^−06^	3.39 × 10^−06^
F7	2.02 × 10^−02^	1.98 × 10^−01^	4.81 × 10^−01^	1.46 × 10^−01^	3.39 × 10^−06^	3.39 × 10^−06^	3.39 × 10^−06^
F8	5.05 × 10^−06^	4.02 × 10^−05^	3.39 × 10^−06^	3.39 × 10^−06^	3.39 × 10^−06^	3.39 × 10^−06^	3.39 × 10^−06^
F9	NaN	NaN	2.54 × 10^−06^	NaN	6.87 × 10^−07^	1.64 × 10^−02^	6.87 × 10^−07^
F10	NaN	NaN	6.87 × 10^−07^	NaN	6.87 × 10^−07^	2.10 × 10^−04^	6.87 × 10^−07^
F11	NaN	NaN	6.87 × 10^−07^	NaN	6.87 × 10^−07^	1.64 × 10^−01^	6.87 × 10^−07^
F12	5.74 × 10^−05^	3.39 × 10^−06^	3.39 × 10^−06^	2.79 × 10^−02^	3.39 × 10^−06^	3.39 × 10^−06^	3.39 × 10^−06^
F13	3.39 × 10^−06^	3.39 × 10^−06^	3.39 × 10^−06^	5.74 × 10^−05^	3.39 × 10^−06^	3.39 × 10^−06^	3.39 × 10^−06^
F14	2.19 × 10^−06^	2.19 × 10^−06^	2.18 × 10^−06^	2.19 × 10^−06^	1.23 × 10^−03^	2.19 × 10^−06^	2.19 × 10^−06^
F15	7.72 × 10^−01^	1.99 × 10^−01^	1.25 × 10^−01^	4.64 × 10^−02^	1.28 × 10^−02^	5.90 × 10^−01^	1.89 × 10^−04^
F16	3.37 × 10^−06^	3.37 × 10^−06^	3.37 × 10^−06^	3.37 × 10^−06^	7.72 × 10^−04^	3.37 × 10^−06^	3.37 × 10^−06^
F17	3.37 × 10^−06^	3.37 × 10^−06^	3.37 × 10^−06^	3.37 × 10^−06^	2.41 × 10^−04^	3.37 × 10^−06^	3.37 × 10^−06^
F18	1.35 × 10^−01^	7.72 × 10^−01^	5.07 × 10^−01^	7.72 × 10^−01^	3.69 × 10^−03^	7.72 × 10^−01^	7.72 × 10^−01^
F19	3.39 × 10^−06^	3.39 × 10^−06^	3.39 × 10^−06^	3.39 × 10^−06^	2.79 × 10^−05^	3.39 × 10^−06^	3.39 × 10^−06^
F20	3.69 × 10^−03^	3.69 × 10^−03^	3.10 × 10^−02^	3.69 × 10^−03^	5.45 × 10^−03^	8.97 × 10^−03^	3.39 × 10^−06^
F21	3.39 × 10^−06^	3.39 × 10^−06^	3.39 × 10^−06^	3.39 × 10^−06^	3.62 × 10^−01^	3.39 × 10^−06^	3.39 × 10^−06^
F22	3.39 × 10^−06^	3.39 × 10^−06^	3.39 × 10^−06^	3.39 × 10^−06^	5.45 × 10^−03^	3.39 × 10^−06^	3.39 × 10^−06^
F23	3.39 × 10^−06^	3.39 × 10^−06^	3.39 × 10^−06^	3.39 × 10^−06^	5.45 × 10^−03^	3.39 × 10^−06^	3.39 × 10^−06^

**Table 7:** The segmented images obtained by ESMA.

Image	nTh = 4	nTh = 6	nTh = 8	nTh = 10
Lena	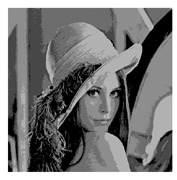	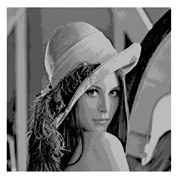	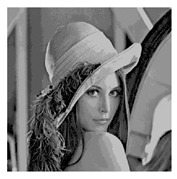	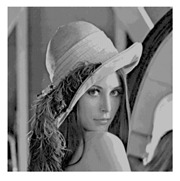
	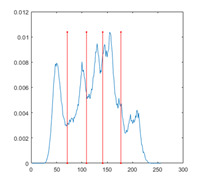	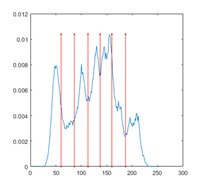	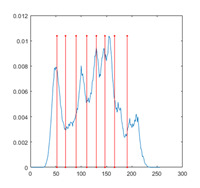	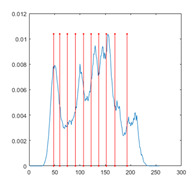
Baboon	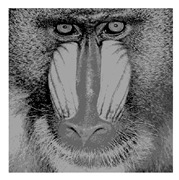	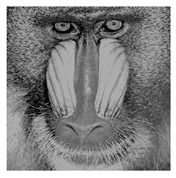	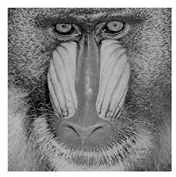	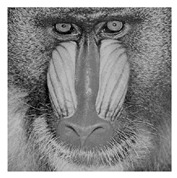
	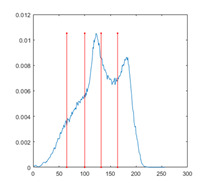	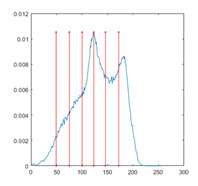	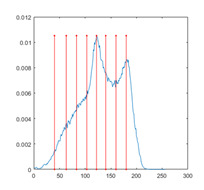	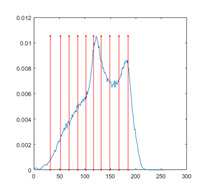
Butterfly	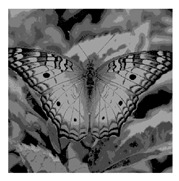	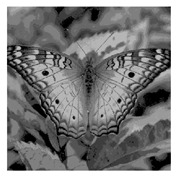	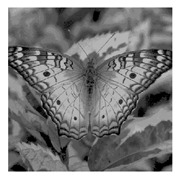	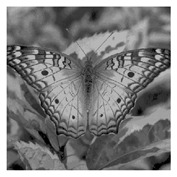
	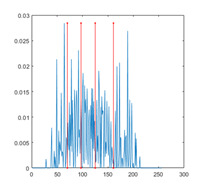	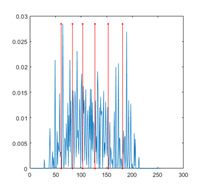	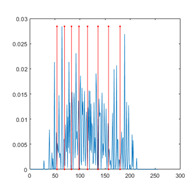	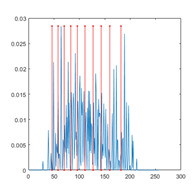
Peppers	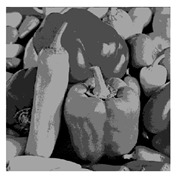	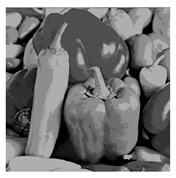	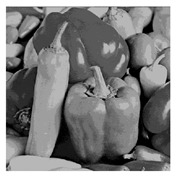	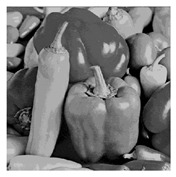
	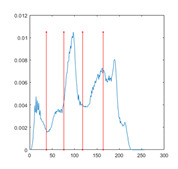	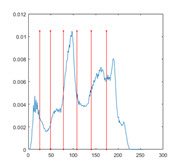	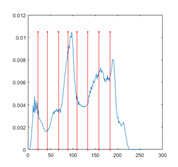	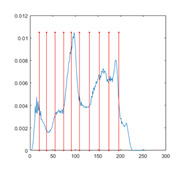
Tank	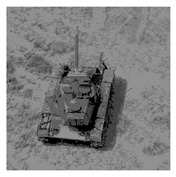	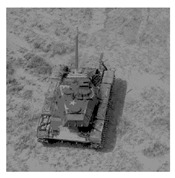	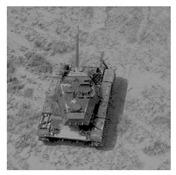	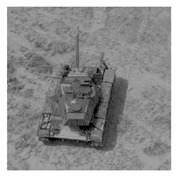
	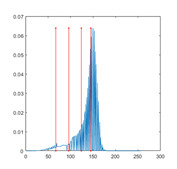	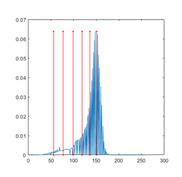	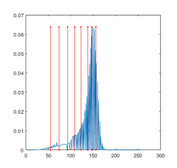	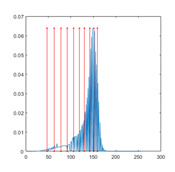
House	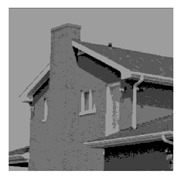	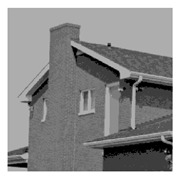	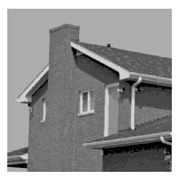	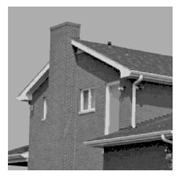
	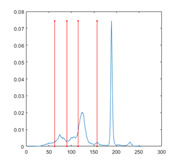	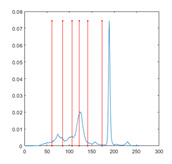	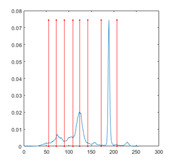	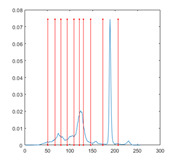
Cameraman	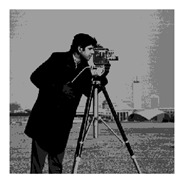	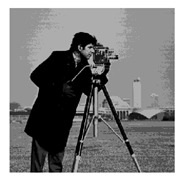	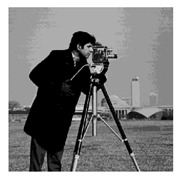	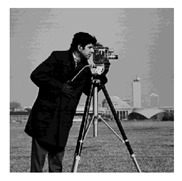
	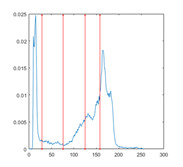	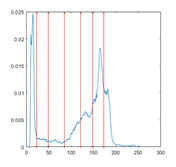	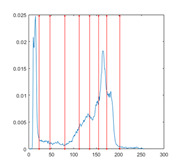	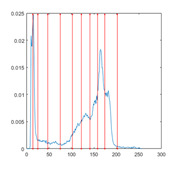
Pirate	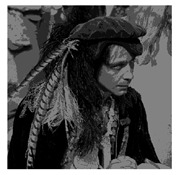	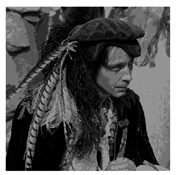	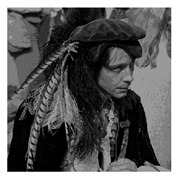	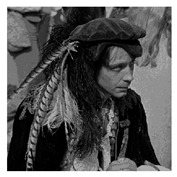
	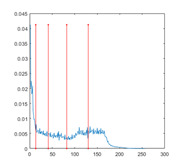	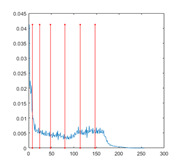	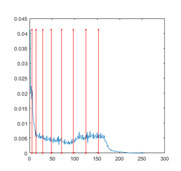	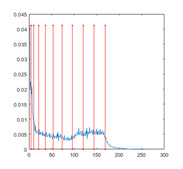

## Data Availability

The data presented in this study are available on request from the corresponding author.

## Appendix A

**Table A1:** The best thresholds obtained by algorithms.

Image	nTh	ESMA	SMA	ROA	AOA	AO	SSA	WOA	SCA
Lena	4	71 109 141 177	71 109 141 177	71 109 141 177	78 112 147 200	71 109 141 177	71 109 141 177	71 109 141 177	78 105 142 181
	6	60 86 113 137 160 187	60 85 112 137 160 187	60 86 113 137 160 187	17 47 53 91 134 176	60 86 113 137 160 187	60 86 113 137 160 187	60 86 113 137 160 187	58 87 105 136 153 186
	8	52 69 90 111 130 147 166 191	50 65 84 102 121 142 163 189	2 52 70 93 116 139 161 188	62 87 109 122 142 164 182 189	52 69 90 111 130 147 166 191	52 69 90 111 130 147 166 191	52 69 90 111 130 147 166 191	1 53 76 101 121 137 165 189
	10	48 60 75 91 107 122 137 152 169 193	50 65 83 100 117 134 149 165 184 203	47 59 73 90 106 121 137 152 169 193	17 45 55 68 78 110 141 155 170 201	3 50 64 82 99 116 134 151 169 193	49 62 78 95 110 126 141 155 172 194	2 50 65 83 100 117 135 151 169 193	1 47 50 71 77 92 110 138 164 187
Baboon	4	65 100 132 164	64 99 131 164	65 100 132 164	47 92 141 190	65 100 132 164	65 100 132 164	65 100 132 164	61 98 134 169
	6	49 75 100 123 146 172	47 73 98 121 145 172	49 75 100 123 146 172	46 69 102 142 179 179	49 75 100 123 146 172	49 75 100 123 146 172	49 75 100 123 146 172	38 56 83 114 135 158
	8	40 63 83 103 122 140 160 180	34 55 74 94 114 133 154 177	39 61 81 101 119 137 158 179	70 94 118 154 157 184 190 194	38 61 81 101 119 137 158 179	39 62 82 102 121 139 159 180	39 61 81 101 119 137 158 179	1 1 26 59 88 113 137 175
	10	32 52 69 86 102 117 132 149 167 185	25 46 62 79 96 113 129 146 164 182	9 40 59 77 95 112 128 145 164 183	28 41 64 89 114 125 156 180 200 229	35 56 74 92 110 127 144 163 182 253	35 56 74 92 110 127 144 163 182 244	8 40 59 77 95 112 128 145 164 183	1 2 2 43 72 91 116 130 146 170
Butterfly	4	70 97 125 161	70 97 125 161	70 97 125 161	69 108 147 226	70 97 125 161	70 97 125 161	70 97 125 161	64 90 119 163
	6	61 83 103 127 153 180	61 83 103 127 153 181	61 83 103 127 153 180	42 66 75 96 135 154	61 82 103 127 153 180	61 82 103 127 153 180	61 82 103 127 153 181	1 62 85 111 136 169
	8	54 69 82 98 115 136 158 181	54 69 82 98 115 136 157 181	54 69 82 98 115 136 158 181	27 52 80 115 130 151 161 233	54 69 82 98 115 135 158 181	54 69 84 100 115 135 157 180	50 69 83 99 115 135 158 181	1 47 74 96 114 138 164 183
	10	26 54 69 83 96 111 127 143 160 182	31 50 68 83 96 111 127 142 160 182	26 54 69 83 96 111 127 143 160 182	35 44 56 57 66 91 104 136 158 202	2 44 57 70 84 100 115 135 158 180	12 54 69 83 96 111 127 142 160 182	33 54 66 82 97 112 127 142 161 182	1 55 61 68 88 105 112 128 149 174
Peppers	4	37 76 118 164	37 77 119 165	37 77 119 165	53 61 109 142	37 77 119 165	37 77 119 165	37 77 119 165	35 72 118 168
	6	25 49 78 108 140 174	32 62 88 115 146 177	25 49 78 108 140 174	13 41 59 102 152 176	24 48 78 108 140 174	25 49 78 108 140 174	25 49 78 108 140 174	1 36 78 114 141 169
	8	22 43 68 89 109 133 158 183	22 42 67 88 108 133 158 183	22 42 67 88 108 133 158 183	30 45 61 73 85 130 172 217	13 45 78 91 124 151 166 202	23 44 71 93 118 148 178 235	22 43 68 89 109 134 158 183	6 37 58 84 101 129 157 180
	10	20 36 55 74 91 109 131 153 174 196	16 26 41 59 77 94 113 137 160 184	11 26 45 62 87 97 122 141 174 199	2 17 30 71 83 98 125 147 152 205	17 43 72 80 102 126 148 157 167 204	22 42 67 87 106 128 151 173 195 236	2 22 42 67 87 106 128 151 173 195	1 1 20 31 55 83 110 136 168 250
Tank	4	67 96 124 145	67 96 123 145	67 96 123 145	57 112 132 147	67 96 124 146	68 98 126 147	67 96 124 146	71 103 126 146
	6	56 77 99 119 136 151	1 64 91 115 135 151	56 77 98 119 136 151	78 92 128 146 175 213	56 77 98 118 135 150	56 77 99 119 136 149	55 77 99 118 136 151	14 63 91 115 131 147
	8	55 74 93 109 123 138 147 156	52 71 90 106 122 135 147 156	2 55 76 95 114 128 141 152	50 89 119 126 150 196 200 241	1 3 56 77 99 118 136 149	55 76 95 114 129 142 152 251	54 75 93 111 127 139 149 159	1 1 51 72 94 119 128 149
	10	47 63 78 92 106 119 130 142 151 159	1 3 52 71 87 103 118 133 145 157	28 55 72 88 102 116 129 140 150 159	15 26 48 67 78 108 137 143 162 224	6 31 57 78 98 119 136 151 212 217	55 76 95 113 129 141 153 211 220 255	43 55 73 88 100 116 129 139 151 158	1 18 35 51 67 93 106 123 146 155
House	4	63 90 115 157	63 90 115 157	63 90 115 157	63 104 161 217	63 90 115 157	63 90 115 157	63 90 115 157	60 85 116 154
	6	61 85 106 122 141 173	63 89 113 138 170 207	63 89 113 138 170 207	33 66 88 114 137 156	63 89 113 138 170 207	63 89 113 138 170 207	63 89 113 138 170 207	2 68 97 115 156 218
	8	55 72 90 109 124 142 172 207	57 75 92 110 124 142 172 207	55 72 90 109 124 142 172 207	6 38 76 97 141 162 180 214	55 73 91 110 124 142 172 207	12 59 78 96 116 138 170 207	55 72 90 109 124 142 172 207	1 1 65 95 118 135 162 207
	10	51 67 80 94 109 121 130 146 173 207	2 51 66 80 95 111 125 143 172 207	6 51 67 81 96 112 125 143 172 207	57 76 94 102 124 144 165 169 182 221	32 51 67 81 95 112 125 143 172 207	13 55 72 90 109 124 142 172 207 244	55 72 90 110 124 142 171 189 199 218	1 58 80 90 109 131 150 184 205 224
Cameraman	4	29 76 125 158	29 76 125 158	29 76 125 158	16 40 91 140	29 76 125 158	29 76 125 158	29 76 125 158	27 78 135 167
	6	23 49 85 121 148 173	23 49 85 121 148 173	23 49 85 121 148 173	7 21 43 78 116 153	23 49 85 121 148 173	23 49 85 121 148 173	23 48 85 120 148 173	21 43 93 124 149 175
	8	23 47 80 112 135 155 173 202	15 26 50 83 115 138 158 177	23 47 80 112 135 155 173 202	23 52 105 112 129 148 161 172	23 47 80 112 134 155 173 202	15 26 50 82 114 137 157 177	23 48 81 112 135 155 173 202	1 1 20 45 86 124 147 171
	10	14 25 47 75 102 122 141 158 174 202	14 23 39 60 88 116 137 156 173 202	14 21 34 56 86 115 137 156 173 202	33 53 76 91 141 159 168 241 250 253	14 28 52 80 105 123 141 156 172 200	14 25 49 82 113 135 155 173 197 230	14 23 39 61 89 117 138 157 174 202	1 15 20 38 57 91 127 145 163 219
Pirate	4	13 41 82 130	13 41 82 130	13 41 82 130	7 21 58 95	13 41 82 130	13 41 82 130	13 41 82 130	13 41 81 124
	6	8 24 48 80 114 147	8 24 48 80 114 147	8 24 49 81 115 148	19 70 97 103 157 254	8 24 49 81 115 148	8 24 48 80 114 147	8 24 49 81 115 148	8 21 50 84 122 162
	8	5 14 29 48 71 97 125 153	5 14 30 49 72 98 125 153	5 13 27 46 68 94 123 152	12 35 54 68 94 142 157 164	5 14 29 48 71 97 125 153	5 15 33 55 83 113 140 170	7 20 41 66 96 126 154 223	4 12 15 24 47 82 114 148
	10	4 10 21 36 53 73 96 120 144 169	4 9 17 28 42 60 81 105 130 156	3 8 16 28 43 61 82 106 130 156	8 28 41 67 98 126 137 145 151 162	4 10 21 36 55 77 101 127 155 240	5 14 29 49 71 96 122 148 177 254	5 14 29 49 72 97 123 148 183 210	1 4 12 29 41 71 83 114 136 163

**Table A2:** The fitness values obtained by algorithms.

Image	nTh	ESMA		SMA		ROA		AOA		AO		SSA		WOA		SCA	
		Mean	Std	Mean	Std	Mean	Std	Mean	Std	Mean	Std	Mean	Std	Mean	Std	Mean	Std
Lena	4	**0.4611**	0	**0.4611**	0	**0.4611**	0	0.7091	0.1235	**0.4611**	0	**0.4611**	0	0.4774	0.0621	0.513	0.0796
	6	**0.245**	0.0063	0.249	0.0142	0.2481	0.004	0.4945	0.0972	0.2567	0.0264	0.2473	0.0045	0.2481	0.014	0.3337	0.0464
	8	**0.1512**	0.0017	0.1634	0.0174	0.1569	0.0019	0.3348	0.063	0.1557	0.0127	0.1624	0.0169	0.1556	0.0127	0.243	0.0337
	10	**0.1048**	0.0015	0.1199	0.0169	0.1093	0.0087	0.2594	0.0366	0.1083	0.0081	0.113	0.01	0.1122	0.0119	0.1921	0.0238
Baboon	4	**0.4962**	0	**0.4962**	0	**0.4962**	0	0.7593	0.1236	**0.4962**	0	**0.4962**	0	**0.4962**	0	0.5342	0.0598
	6	**0.2781**	0.0001	0.2785	0.0003	0.2782	0	0.4957	0.0791	0.281	0.0151	0.2783	0.0001	0.2806	0.0131	0.3507	0.0404
	8	**0.178**	0.0004	0.1805	0.0054	0.1784	0.0037	0.3544	0.0477	0.1789	0.0073	0.1868	0.0165	0.1783	0.004	0.2683	0.0396
	10	**0.1229**	0.0004	0.1293	0.0075	0.123	0.0017	0.2625	0.0311	0.1248	0.0071	0.1387	0.0149	0.1256	0.0081	0.2136	0.0275
Butterfly	4	**0.3968**	0	**0.3968**	0	**0.3968**	0	0.7151	0.1365	**0.3968**	0	**0.3968**	0	0.4116	0.0561	0.4669	0.0886
	6	**0.229**	0.0043	0.2297	0.0184	0.2348	0.0278	0.4595	0.0859	0.229	0.0134	0.2292	0.0135	0.2372	0.0298	0.3061	0.0367
	8	**0.1356**	0.0141	0.1413	0.0181	0.1385	0.0224	0.305	0.0517	0.1389	0.007	0.1383	0.0157	0.138	0.0165	0.2219	0.0258
	10	**0.0853**	0.0039	0.1069	0.0157	0.0923	0.0097	0.244	0.0463	0.0926	0.0088	0.1059	0.0151	0.0969	0.015	0.1774	0.0244
Peppers	4	**0.704**	0	**0.704**	0	**0.704**	0	1.0897	0.1784	**0.704**	0	**0.704**	0	**0.704**	0	0.7277	0.015
	6	0.4019	0.0027	0.4007	0.0019	**0.3997**	0.0003	0.6925	0.0853	0.3998	0.0003	0.4002	0.0013	**0.3997**	0.0001	0.4913	0.0585
	8	**0.2456**	0.0001	0.2481	0.0059	0.246	0.0025	0.4845	0.067	0.2459	0.0001	0.256	0.0237	0.2459	0.0001	0.3663	0.0361
	10	**0.1755**	0.0057	0.1779	0.0128	0.1793	0.0003	0.3631	0.0607	0.1792	0.0001	0.1931	0.0196	0.1792	0.0002	0.2913	0.0317
Tank	4	**0.1992**	0.0001	0.1993	0.0001	**0.1992**	0	0.3468	0.0542	**0.1992**	0	**0.1992**	0.0001	0.2026	0.0182	0.2184	0.0292
	6	**0.106**	0.0012	0.1153	0.015	0.1069	0.0002	0.2579	0.0519	0.1127	0.0136	0.1171	0.0161	0.1106	0.0114	0.1694	0.0246
	8	**0.0707**	0.0022	0.0816	0.0148	0.0797	0.0078	0.1962	0.0468	0.0709	0.0058	0.0774	0.0089	0.0726	0.0092	0.1395	0.0196
	10	**0.045**	0.0048	0.0655	0.0126	0.049	0.006	0.1462	0.029	0.0524	0.0072	0.0612	0.0098	0.0521	0.0063	0.1024	0.0173
House	4	**0.3302**	0.0093	**0.3302**	0	0.3345	0.0237	0.478	0.0713	**0.3302**	0	**0.3302**	0	**0.3302**	0.0001	0.3512	0.0313
	6	0.1816	0.0245	**0.1606**	0	0.1658	0.0197	0.3072	0.0429	0.1634	0.0153	0.1632	0.0142	0.1632	0.0142	0.2239	0.0329
	8	**0.0964**	0.0127	0.1018	0.0131	0.1009	0.0155	0.2271	0.034	0.0966	0.0078	0.1031	0.0128	0.1025	0.0134	0.1552	0.0198
	10	**0.0665**	0.0019	0.0773	0.0112	0.0669	0.0028	0.1686	0.0292	0.0705	0.0065	0.0715	0.0053	0.0714	0.0057	0.1246	0.0194
Cameraman	4	**0.5385**	0	**0.5385**	0	**0.5385**	0	0.7752	0.1214	**0.5385**	0	**0.5385**	0	**0.5385**	0	0.5506	0.0067
	6	**0.3032**	0	0.3033	0	0.3033	0.0001	0.5071	0.076	0.3033	0	0.3105	0.0166	0.3033	0.0002	0.3682	0.0478
	8	**0.2031**	0.0042	0.2061	0.0063	0.2077	0.0117	0.3548	0.0569	0.2041	0.0021	0.2046	0.0015	0.2049	0.0087	0.2823	0.0431
	10	**0.1368**	0.0103	0.1396	0.0152	0.139	0.0088	0.2832	0.0426	0.1387	0.0061	0.1427	0.013	0.1383	0.0054	0.2299	0.0209
Pirate	4	**1.0403**	0	**1.0403**	0	**1.0403**	0	1.6838	0.3576	**1.0403**	0	**1.0403**	0	**1.0403**	0	1.0588	0.0117
	6	0.5845	0.0045	0.5822	0.0016	**0.5815**	0	1.1018	0.2407	**0.5815**	0	0.5937	0.0458	**0.5815**	0.0001	0.6456	0.0341
	8	0.3593	0.0023	0.3599	0.0026	**0.3576**	0.0002	0.8182	0.1572	0.3577	0.0004	0.3904	0.0317	**0.3576**	0.0002	0.4814	0.0636
	10	**0.2413**	0.0058	0.2499	0.0056	0.2445	0.0007	0.6187	0.1055	0.2461	0.0095	0.3038	0.023	0.2443	0.0006	0.3821	0.0403

**Table A3:** The PSNR values obtained by algorithms.

Image	nTh	ESMA		SMA		ROA		AOA		AO		SSA		WOA		SCA	
		Mean	Std	Mean	Std	Mean	Std	Mean	Std	Mean	Std	Mean	Std	Mean	Std	Mean	Std
Lena	4	**18.7867**	0	**18.7867**	0	**18.7867**	0	17.9115	0.7829	**18.7867**	0	**18.7867**	0	18.7211	0.257	18.5889	0.4204
	6	**21.1436**	0.3611	20.9155	0.201	20.9023	0.0791	19.6602	1.0424	20.9171	0.2101	20.9881	0.2528	20.888	0.0102	20.7039	0.8201
	8	**23.3637**	0.1453	23.2477	0.5122	23.3548	0.1958	21.3528	1.3934	23.2899	0.2038	23.3507	0.5784	23.3314	0.3472	22.7486	1.2342
	10	**25.3269**	0.329	24.9085	0.8677	25.255	0.5987	22.5184	1.6544	25.0865	0.5052	24.667	0.4771	25.3044	0.6412	23.8616	1.4136
Baboon	4	**20.7335**	0.0247	**20.7335**	0.0247	20.7215	0.0157	18.8128	1.0221	20.7215	0.0157	20.7163	0	20.7198	0.0131	20.4913	0.5255
	6	**24.195**	0.0307	24.1869	0.0354	24.1523	0	21.2006	0.9268	24.1063	0.2569	24.1673	0.02	24.1101	0.2313	22.936	0.6118
	8	**26.5418**	0.0426	26.4394	0.2024	26.5412	0.1283	22.8569	0.9407	26.5353	0.1962	26.3104	0.4386	26.5417	0.1499	24.4069	0.707
	10	**28.3939**	0.07	27.9523	0.3452	28.3236	0.1161	24.3764	0.6605	28.2776	0.2594	27.7754	0.5268	28.2071	0.3548	25.566	0.6591
Butterfly	4	**19.384**	0	**19.384**	0	**19.384**	0	17.3819	1.7028	**19.384**	0	19.3918	0.0237	19.3124	0.2727	18.8569	0.8241
	6	**23.027**	0.3381	22.6981	0.3791	22.4712	0.1428	20.1249	1.5871	22.4572	0.1907	22.7495	0.4085	22.4194	0.3011	21.7811	1.2288
	8	25.2782	0.4232	25.1357	0.5241	25.281	0.464	22.6981	1.1545	25.2233	0.2508	25.0719	0.5173	**25.6065**	0.5072	23.4691	0.959
	10	27.8053	0.9404	26.9718	1.0735	27.7339	0.9339	23.6315	1.6794	26.9143	1.088	26.6992	1.2001	**27.8357**	0.9296	24.9351	1.0205
Peppers	4	**20.3048**	0	20.2961	0.0175	**20.3048**	0	18.4579	1.0843	**20.3048**	0	20.3033	0.0079	**20.3048**	0	20.1694	0.2803
	6	**23.1363**	0.1851	23.0465	0.131	22.9841	0.0193	20.6058	0.9365	22.9847	0.0241	23.0143	0.097	22.9755	0.0182	22.1766	0.5925
	8	**25.4398**	0.0251	25.3289	0.2152	25.4282	0.0478	22.2705	0.9964	25.4386	0.0236	25.2324	0.4713	25.4277	0.0225	23.3841	0.5773
	10	26.7164	0.213	26.6864	0.3272	26.9926	0.0468	23.7216	1.1374	**27.0096**	0.0336	26.5867	0.4785	26.986	0.0382	24.3768	0.5042
Tank	4	23.621	0.1847	23.5904	0.1884	23.6233	0.1601	21.0197	1.3991	**23.631**	0.1665	23.619	0.1653	23.5073	0.4685	23.1379	0.8407
	6	**27.1319**	0.1967	26.5793	0.8502	27.1103	0.1303	22.6586	1.6433	26.734	0.7977	26.4843	0.9135	26.9133	0.5067	24.8651	0.8758
	8	**29.1754**	0.3681	28.6313	0.9286	28.6987	0.3745	24.7671	1.168	28.6371	0.375	28.55	0.6137	28.6097	0.4403	26.1582	1.0087
	10	**31.0145**	0.325	29.9936	0.8134	30.9248	0.7471	25.8087	1.3187	30.1609	0.6403	29.7464	1.014	30.8067	0.5992	28.0394	1.0181
House	4	**19.6568**	0	**19.6568**	0	19.6148	0.2299	18.4479	1.8064	**19.6568**	0	**19.6568**	0	19.6602	0.0129	19.3939	0.6208
	6	**22.8143**	0.1219	22.7241	0.0352	22.5672	0.5813	21.1436	1.3549	22.6941	0.0906	22.6359	0.4089	22.6515	0.4135	21.6748	1.216
	8	**24.6994**	0.0803	24.4874	0.4175	24.6165	0.3892	22.5041	1.6702	24.642	0.2449	24.4491	0.4135	24.6646	0.2606	24.1296	1.4269
	10	25.9749	0.1114	25.6998	0.3883	26.0617	0.1936	23.7466	1.467	**26.0764**	0.5714	25.8552	0.4539	26.0151	0.2245	24.7753	1.5695
Cameraman	4	**21.4059**	0	**21.4059**	0	**21.4059**	0	19.2516	1.3089	**21.4059**	0	**21.4059**	0	21.4021	0.0142	21.1921	0.4044
	6	23.905	0	23.9124	0.019	23.911	0.0177	21.3045	1.2432	23.9102	0.0178	23.8265	0.1787	**23.9187**	0.0413	22.9911	0.8038
	8	25.5199	0.4548	25.411	0.4505	25.5124	0.4735	23.1434	1.1121	**25.5978**	0.395	25.7295	0.315	25.6113	0.4191	24.1015	0.7116
	10	**27.5098**	0.3286	27.1335	0.5009	27.1949	0.439	24.3376	1.1574	27.487	0.3557	27.3671	0.4647	27.303	0.2443	24.9613	0.8164
Pirate	4	**20.9183**	0	**20.9183**	0	**20.9183**	0	19.2525	1.2367	**20.9183**	0	**20.9183**	0	**20.9183**	0	20.8557	0.2473
	6	23.7017	0.2661	23.8158	0.0891	23.8575	0	21.3707	1.4619	23.8542	0.0126	23.7243	0.3979	**23.8606**	0.0172	22.846	0.5803
	8	25.7016	0.2009	25.5707	0.233	**25.7204**	0.0341	22.6917	1.4888	25.7117	0.07	25.4364	0.4623	25.7148	0.0309	24.092	0.7173
	10	**27.1522**	0.3278	27.0123	0.2586	27.1135	0.0535	23.5524	1.3516	27.112	0.1902	26.5997	0.3443	27.1225	0.0353	25.0381	0.6051

**Table A4:** The SSIM values obtained by algorithms.

Image	nTh	ESMA		SMA		ROA		AOA		AO		SSA		WOA		SCA	
		Mean	Std	Mean	Std	Mean	Std	Mean	Std	Mean	Std	Mean	Std	Mean	Std	Mean	Std
Lena	4	**0.649**	0	**0.649**	0	**0.649**	0	0.6311	0.0414	**0.649**	0	**0.649**	0	0.6484	0.0045	0.6465	0.0112
	6	**0.7284**	0.0077	0.7232	0.0049	0.7236	0.0033	0.6904	0.0484	0.7236	0.0047	0.725	0.0055	0.723	0.0007	0.7131	0.0239
	8	**0.7814**	0.0025	0.779	0.0126	0.7812	0.004	0.7327	0.0463	0.7793	0.0044	0.7813	0.0145	**0.7814**	0.0082	0.7656	0.031
	10	0.8208	0.007	0.8158	0.0174	**0.8256**	0.0103	0.7652	0.0474	0.8223	0.0088	0.8115	0.0104	0.8252	0.0115	0.7935	0.0352
Baboon	4	**0.8041**	0.0002	**0.8041**	0.0002	**0.8041**	0.0001	0.7359	0.0338	**0.8041**	0.0001	**0.8041**	0	**0.8041**	0.0001	0.7937	0.0159
	6	**0.8766**	0.0006	0.8764	0.0011	0.8762	0	0.8052	0.0255	0.8752	0.005	0.8761	0.0005	0.8754	0.0043	0.8511	0.0127
	8	**0.917**	0.0012	0.9144	0.0029	0.9158	0.0017	0.8461	0.0232	0.9157	0.0028	0.9125	0.0062	0.916	0.0021	0.8806	0.0124
	10	**0.9395**	0.0013	0.9351	0.0045	0.9388	0.0013	0.8778	0.0116	0.939	0.003	0.933	0.0068	0.9381	0.0043	0.8992	0.0124
Butterfly	4	**0.6746**	0	**0.6746**	0	**0.6746**	0	0.589	0.069	**0.6746**	0	0.6745	0.0003	0.6721	0.0094	0.6512	0.0318
	6	**0.786**	0.0076	0.7779	0.0086	0.7737	0.0038	0.6924	0.0584	0.7734	0.0051	0.7796	0.01	0.7719	0.0085	0.748	0.0329
	8	**0.8496**	0.0056	0.8438	0.0125	0.8474	0.0112	0.777	0.0309	**0.8496**	0.0053	0.8437	0.0119	0.8525	0.0081	0.7987	0.0223
	10	**0.8996**	0.0115	0.8816	0.0175	0.897	0.0129	0.8022	0.037	0.8866	0.015	0.8776	0.019	0.8963	0.0139	0.8325	0.0205
Peppers	4	**0.6714**	0.0007	0.6717	0.0006	**0.6714**	0	0.632	0.0293	**0.6714**	0	0.6715	0.0003	**0.6714**	0	0.6699	0.0062
	6	0.7371	0.0048	0.7397	0.0033	0.7411	0.0005	0.6915	0.024	0.7413	0.0004	0.7403	0.0026	**0.7415**	0.0005	0.7271	0.0153
	8	**0.7873**	0.0006	0.7867	0.0019	0.7872	0.0004	0.7291	0.0246	0.787	0.0006	0.7823	0.0107	0.787	0.0005	0.7623	0.0131
	10	**0.8231**	0.0011	0.8213	0.0037	0.8224	0.0007	0.7613	0.0274	0.8226	0.0004	0.8099	0.0107	0.8226	0.0006	0.7836	0.0139
Tank	4	**0.777**	0.0033	0.7759	0.0039	0.7756	0.0033	0.6936	0.0404	0.7741	0.0044	0.7759	0.0041	0.7728	0.0124	0.7632	0.0248
	6	0.8682	0.0036	0.8601	0.014	**0.8694**	0.0034	0.7351	0.0509	0.8631	0.0137	0.8584	0.0152	0.8656	0.0098	0.8027	0.0257
	8	**0.9206**	0.0049	0.8965	0.0163	0.9108	0.0089	0.7926	0.0373	0.9072	0.0086	0.8999	0.011	0.9074	0.0096	0.8406	0.0199
	10	0.9307	0.0077	0.9153	0.0134	**0.9338**	0.0074	0.8221	0.0371	0.9275	0.0102	0.9188	0.0118	0.931	0.0073	0.8763	0.0234
House	4	**0.7912**	0	**0.7912**	0	0.7896	0.0083	0.735	0.0517	**0.7912**	0	**0.7912**	0	**0.7912**	0.0009	0.7798	0.0199
	6	**0.8424**	0.0088	0.8354	0.0008	0.8339	0.005	0.7814	0.0527	0.8349	0.0016	0.8345	0.0032	0.8348	0.0034	0.8218	0.0174
	8	**0.8904**	0.0011	0.8848	0.0116	0.8875	0.0114	0.8289	0.029	0.889	0.0067	0.8823	0.0126	0.888	0.0071	0.8591	0.0131
	10	**0.9205**	0.0033	0.9129	0.0093	0.920	0.0035	0.8466	0.0328	0.9171	0.0059	0.9142	0.0069	0.9193	0.0055	0.8778	0.019
Cameraman	4	**0.6955**	0	**0.6955**	0	**0.6955**	0	0.6788	0.0488	**0.6955**	0	**0.6955**	0	0.6954	0.0003	0.6897	0.0167
	6	**0.7361**	0	**0.7361**	0.0003	**0.7361**	0.0003	0.7071	0.0263	**0.7361**	0.0003	0.7334	0.0061	**0.7361**	0.0008	0.7254	0.0164
	8	0.787	0.0221	0.786	0.0193	**0.7883**	0.0218	0.7477	0.0387	0.7799	0.0176	0.7686	0.0104	0.7756	0.0176	0.7715	0.0321
	10	**0.8412**	0.0065	0.8364	0.0101	0.8395	0.007	0.7831	0.0548	0.8398	0.0103	0.823	0.0236	0.8395	0.0081	0.8193	0.0343
Pirate	4	**0.6868**	0	**0.6868**	0	**0.6868**	0	0.6198	0.0332	**0.6868**	0	**0.6868**	0	**0.6868**	0	0.6841	0.0043
	6	**0.7765**	0.0027	0.7759	0.0015	0.7762	0	0.6947	0.0318	0.7762	0	0.7736	0.01	0.7761	0.0005	0.7723	0.0084
	8	0.8421	0.0026	0.8419	0.0021	**0.8435**	0.0003	0.7365	0.0281	0.8434	0.0006	0.8301	0.0111	**0.8435**	0.0003	0.8173	0.0133
	10	0.8746	0.0011	0.8748	0.0016	0.8761	0.0007	0.7752	0.0232	0.8757	0.0027	0.8571	0.0069	**0.8762**	0.0006	0.842	0.0102

**Table A5:** The FSIM values obtained by algorithms.

Image	nTh	ESMA		SMA		ROA		AOA		AO		SSA		WOA		SCA	
		Mean	Std	Mean	Std	Mean	Std	Mean	Std	Mean	Std	Mean	Std	Mean	Std	Mean	Std
Lena	4	**0.855**	0	**0.855**	0	**0.855**	0	0.8215	0.0183	**0.855**	0	**0.855**	0	0.8531	0.0075	0.8495	0.0119
	6	0.8933	0.0131	0.8999	0.0074	0.9013	0.004	0.8535	0.0181	0.8979	0.009	0.8987	0.0093	**0.9017**	0.0031	0.8765	0.0089
	8	**0.9100**	0.0008	0.9068	0.007	0.9091	0.0019	0.8791	0.0179	0.9079	0.0025	0.9096	0.0078	0.9096	0.0046	0.8974	0.0113
	10	0.9233	0.0012	0.9233	0.0097	0.9258	0.0087	0.8947	0.0187	0.924	0.0062	0.9218	0.0037	**0.9273**	0.0092	0.9115	0.0157
Baboon	4	**0.9268**	0.0004	**0.9268**	0.0004	0.9266	0.0003	0.8948	0.0222	0.9266	0.0003	0.9265	0	0.9266	0.0002	0.9226	0.0108
	6	**0.9602**	0.0005	**0.9602**	0.0009	0.9591	0	0.9248	0.0192	0.9587	0.0025	0.9597	0.0006	0.9587	0.0022	0.9473	0.0076
	8	0.9769	0.0011	0.9766	0.0015	**0.9771**	0.0005	0.9445	0.0144	0.9771	0.0012	0.976	0.0027	0.977	0.0008	0.9613	0.0098
	10	0.9859	0.0006	0.9851	0.0016	**0.9861**	0.0006	0.9576	0.0135	**0.9861**	0.0011	0.984	0.0023	0.9857	0.0015	0.9684	0.007
Butterfly	4	**0.8454**	0	**0.8454**	0	**0.8454**	0	0.7915	0.0257	**0.8454**	0	**0.8454**	0	0.8433	0.008	0.832	0.018
	6	**0.902**	0.0012	0.9006	0.0048	0.8996	0.0052	0.8441	0.029	0.9008	0.0046	0.9006	0.0047	0.8985	0.0081	0.8789	0.015
	8	0.9352	0.004	0.9344	0.0061	0.9363	0.0054	0.8881	0.0178	0.9365	0.0029	0.9344	0.0057	**0.9397**	0.0056	0.9079	0.0129
	10	0.9615	0.0083	0.9538	0.01	0.9613	0.008	0.9029	0.0227	0.9535	0.0097	0.9516	0.0109	**0.9618**	0.0084	0.9254	0.0129
Peppers	4	**0.849**	0	**0.849**	0	**0.849**	0	0.8141	0.0181	**0.849**	0	**0.849**	0	**0.849**	0	0.8465	0.0032
	6	**0.8992**	0.0018	0.8983	0.0012	0.8977	0.0002	0.8529	0.0156	0.8977	0.0003	0.898	0.0008	0.8976	0.0003	0.8842	0.01
	8	**0.933**	0.0006	0.931	0.0034	0.9328	0.0004	0.8818	0.0146	0.9329	0.0003	0.9298	0.0069	0.9328	0.0003	0.9039	0.0086
	10	0.9498	0.0068	0.9511	0.0073	**0.9578**	0.0005	0.907	0.0173	**0.9578**	0.0004	0.9532	0.0068	**0.9578**	0.0004	0.9176	0.0099
Tank	4	0.9154	0.0025	**0.9158**	0.0023	0.9153	0.0023	0.8516	0.0258	0.9149	0.0021	0.9154	0.0021	0.9145	0.0079	0.9028	0.0129
	6	0.9506	0.0021	0.9461	0.0087	**0.9508**	0.0022	0.8827	0.031	0.9468	0.0068	0.9446	0.0091	0.9487	0.0056	0.9287	0.0113
	8	**0.9672**	0.0023	0.964	0.0079	0.9657	0.0033	0.9133	0.0192	0.9658	0.0044	0.9631	0.0049	0.9643	0.0038	0.9403	0.0107
	10	**0.9789**	0.0018	0.9751	0.0056	0.9784	0.0041	0.9313	0.0171	0.9743	0.0049	0.9724	0.0073	0.9787	0.0041	0.9556	0.0107
House	4	**0.7969**	0.0027	0.7962	0	0.7954	0.0045	0.7863	0.0214	0.7962	0	0.7962	0	0.7963	0.0006	0.7932	0.0097
	6	0.867	0.0087	**0.8747**	0.0006	0.8728	0.0066	0.8262	0.0219	0.8734	0.0061	0.8736	0.0045	0.8739	0.0046	0.853	0.0159
	8	**0.9104**	0.0052	0.9076	0.0068	0.909	0.0071	0.857	0.0193	0.9101	0.0038	0.9079	0.0058	0.9097	0.0041	0.8883	0.0107
	10	0.9334	0.0018	0.9287	0.0066	0.9326	0.0023	0.8817	0.017	0.9315	0.0043	0.9317	0.0043	**0.9344**	0.0039	0.9019	0.012
Cameraman	4	**0.8546**	0	**0.8546**	0	**0.8546**	0	0.8227	0.0229	**0.8546**	0	**0.8546**	0	**0.8546**	0.0002	0.8506	0.0091
	6	0.9023	0.0023	**0.9028**	0.0003	**0.9028**	0.0002	0.8601	0.0238	0.9027	0.0003	0.9007	0.0045	**0.9028**	0.0005	0.8855	0.0143
	8	0.9211	0.0076	0.9197	0.0088	0.9213	0.009	0.8865	0.0173	0.9237	0.0084	**0.9283**	0.007	0.925	0.0089	0.9004	0.0102
	10	0.9374	0.0037	0.9363	0.005	0.9366	0.0036	0.9037	0.0155	0.9394	0.0031	0.9396	0.0045	**0.94**	0.0018	0.913	0.0102
Pirate	4	**0.8914**	0	**0.8914**	0	**0.8914**	0	0.8501	0.0275	**0.8914**	0	**0.8914**	0	**0.8914**	0	0.8894	0.0046
	6	**0.9419**	0.0039	0.9389	0.0016	0.9417	0	0.8933	0.0302	0.9417	0.0002	0.9396	0.0062	0.9417	0.0001	0.9243	0.0089
	8	**0.9603**	0.002	0.9591	0.0022	0.9602	0.0002	0.9136	0.0266	0.9602	0.0006	0.9612	0.0055	0.9601	0.0002	0.941	0.0102
	10	0.9726	0.0046	0.9737	0.0036	**0.9766**	0.0003	0.928	0.0236	0.9762	0.0021	0.9734	0.0038	0.9765	0.0002	0.9519	0.0073

**Table A6:** The *p*-values obtained by algorithms.

Images	nTh	SMA	ROA	AOA	AO	SSA	WOA	SCA
Lena	4	NaN	NaN	1.22 × 10^−12^	NaN	NaN	3.34 × 10^−01^	1.22 × 10^−12^
	6	4.44 × 10^−02^	4.70 × 10^−04^	1.75 × 10^−11^	3.27 × 10^−02^	6.45 × 10^−02^	1.45 × 10^−01^	1.75 × 10^−11^
	8	3.38 × 10^−05^	8.56 × 10^−02^	2.47 × 10^−11^	8.62 × 10^−01^	3.48 × 10^−02^	1.10 × 10^−01^	2.47 × 10^−11^
	10	1.28 × 10^−08^	7.05 × 10^−03^	2.31 × 10^−11^	4.17 × 10^−01^	1.32 × 10^−04^	1.10 × 10^−01^	2.31 × 10^−11^
Baboon	4	4.45 × 10^−01^	6.55 × 10^−04^	1.34 × 10^−11^	6.55 × 10^−04^	2.56 × 10^−03^	1.28 × 10^−04^	1.34 × 10^−11^
	6	8.44 × 10^−01^	7.04 × 10^−11^	1.89 × 10^−11^	8.74 × 10^−10^	2.63 × 10^−05^	4.80 × 10^−08^	1.89 × 10^−11^
	8	1.48 × 10^−03^	3.11 × 10^−10^	2.75 × 10^−11^	7.26 × 10^−11^	2.89 × 10^−02^	7.37 × 10^−09^	2.75 × 10^−11^
	10	9.75 × 10^−10^	4.05 × 10^−07^	2.70 × 10^−11^	6.81 × 10^−07^	8.33 × 10^−03^	3.24 × 10^−03^	2.70 × 10^−11^
Butterfly	4	NaN	NaN	1.21 × 10^−12^	1.09 × 10^−02^	4.18 × 10^−02^	3.34 × 10^−01^	1.21 × 10^−12^
	6	3.13 × 10^−02^	1.14 × 10^−02^	2.20 × 10^−11^	1.06 × 10^−03^	4.71 × 10^−01^	5.30 × 10^−01^	2.20 × 10^−11^
	8	7.74 × 10^−02^	1.04 × 10^−03^	2.65 × 10^−11^	6.82 × 10^−02^	4.69 × 10^−02^	9.47 × 10^−01^	2.65 × 10^−11^
	10	4.91 × 10^−06^	3.64 × 10^−03^	1.44 × 10^−11^	1.04 × 10^−02^	2.49 × 10^−06^	2.85 × 10^−04^	1.44 × 10^−11^
Peppers	4	5.69 × 10^−01^	5.47 × 10^−03^	7.57 × 10^−12^	5.47 × 10^−03^	5.47 × 10^−03^	5.47 × 10^−03^	7.57 × 10^−12^
	6	5.79 × 10^−01^	2.85 × 10^−01^	1.17 × 10^−11^	1.38 × 10^−01^	4.24 × 10^−02^	1.38 × 10^−01^	1.17 × 10^−11^
	8	4.13 × 10^−03^	3.55 × 10^−01^	1.97 × 10^−11^	1.10 × 10^−01^	9.50 × 10^−01^	1.75 × 10^−01^	1.97 × 10^−11^
	10	4.43 × 10^−04^	7.18 × 10^−04^	2.83 × 10^−11^	2.73 × 10^−02^	7.24 × 10^−05^	8.41 × 10^−04^	2.83 × 10^−11^
Tank	4	5.69 × 10^−01^	7.99 × 10^−01^	7.57 × 10^−12^	1.73 × 10^−01^	3.26 × 10^−01^	4.56 × 10^−02^	7.57 × 10^−12^
	6	4.72 × 10^−02^	5.89 × 10^−01^	3.16 × 10^−12^	8.90 × 10^−03^	4.76 × 10^−02^	1.66 × 10^−04^	3.16 × 10^−12^
	8	6.38 × 10^−08^	1.01 × 10^−03^	2.90 × 10^−11^	1.10 × 10^−01^	4.36 × 10^−02^	3.25 × 10^−02^	2.90 × 10^−11^
	10	5.39 × 10^−06^	1.97 × 10^−02^	2.93 × 10^−11^	9.12 × 10^−01^	4.29 × 10^−05^	5.10 × 10^−01^	2.93 × 10^−11^
House	4	1.61 × 10^−01^	1.61 × 10^−01^	2.37 × 10^−12^	1.61 × 10^−01^	9.86 × 10^−01^	9.59 × 10^−01^	8.38 × 10^−10^
	6	9.78 × 10^−01^	4.80 × 10^−02^	9.36 × 10^−12^	7.68 × 10^−01^	2.78 × 10^−03^	2.31 × 10^−01^	3.09 × 10^−07^
	8	7.83 × 10^−07^	2.43 × 10^−06^	5.21 × 10^−12^	5.90 × 10^−06^	3.32 × 10^−03^	4.98 × 10^−07^	5.21 × 10^−12^
	10	1.55 × 10^−04^	8.42 × 10^−01^	2.85 × 10^−11^	5.54 × 10^−01^	1.06 × 10^−06^	2.22 × 10^−01^	2.85 × 10^−11^
Cameraman	4	NaN	NaN	1.21 × 10^−12^	NaN	NaN	3.34 × 10^−02^	1.21 × 10^−12^
	6	9.59 × 10^−01^	2.05 × 10^−02^	2.36 × 10^−12^	2.04 × 10^−02^	2.95 × 10^−01^	1.66 × 10^−03^	1.69 × 10^−11^
	8	2.87 × 10^−01^	4.52 × 10^−02^	2.66 × 10^−11^	1.40 × 10^−01^	4.12 × 10^−03^	2.50 × 10^−02^	2.66 × 10^−11^
	10	4.89 × 10^−02^	4.55 × 10^−02^	2.85 × 10^−11^	9.88 × 10^−01^	1.41 × 10^−01^	4.46 × 10^−02^	2.85 × 10^−11^
Pirate	4	NaN	NaN	1.22 × 10^−12^	NaN	NaN	NaN	1.22 × 10^−12^
	6	1.38 × 10^−06^	1.89 × 10^−11^	2.83 × 10^−11^	4.22 × 10^−12^	6.65 × 10^−07^	2.73 × 10^−11^	2.83 × 10^−11^
	8	7.02 × 10^−02^	4.15 × 10^−07^	2.93 × 10^−11^	2.38 × 10^−04^	6.34 × 10^−08^	1.67 × 10^−06^	2.93 × 10^−11^
	10	2.80 × 10^−01^	2.47 × 10^−07^	2.95 × 10^−11^	9.18 × 10^−06^	2.32 × 10^−10^	1.82 × 10^−07^	2.95 × 10^−11^
